# Modification of Gene Expression Involved in Alkaloid Production in Opium Poppy by VIGS Combined With Pretreatment of Macerozyme Enzyme

**DOI:** 10.1002/pld3.70034

**Published:** 2025-01-07

**Authors:** Deniz Ece Özcan, Deniz Köm, Ozan Doğan, Semiha Erişen, Selma Onarici

**Affiliations:** ^1^ Climate and Life Sciences TUBITAK Marmara Research Center Kocaeli Turkiye; ^2^ Molecular Biology and Genetics, Institute of Sciences Yildiz Technical University Istanbul Turkiye; ^3^ Poppy and Alkaloid Affairs Department Turkish Grain Board Ankara Turkiye

**Keywords:** alkaloid production, macerozyme enzyme, morphine biosynthesis, *Papaver somniferum* L, quantitative reverse transcription PCR, somatic embryo suspension cultures, virus‐induced gene silencing (VIGS)

## Abstract

*Papaver somniferum* L., a medicinal plant renowned for its pharmaceutical alkaloids, has captivated scientific interest due to its rich secondary metabolite profile. This study explores a novel approach to manipulating alkaloid biosynthesis pathways by integrating virus‐induced gene silencing (VIGS) with macerozyme enzyme pretreatment. Targeting key genes in the benzylisoquinoline alkaloid (BIA) pathway (*CODM*, *T6ODM*, *COR*, *DIOX2*), the research aimed to elucidate the transformative potential of enzymatic preconditioning in somatic embryo cultures. To address the cell wall barrier, a known limitation in genetic manipulation, macerozyme pretreatment was employed, significantly enhancing gene silencing efficacy. Quantitative reverse transcription PCR analyses revealed significant alterations in gene expression profiles with macerozyme pretreatment, whereas no changes were observed in its absence. The T6ODM + DIOX combination was the most effective, reducing *CODM*, *T6ODM*, and *DIOX2* expression by 72%, 65%, and 60%, respectively. Conversely, *T6ODM* expression increased by up to 107% in the CODM treatment. Notably, *COR* expression displayed dual regulatory dynamics, with suppression (47% decrease in T6ODM + DIOX) and enhancement (49% increase in CODM+DIOX) observed under different conditions. These findings underscore the complex interplay of gene regulation in the morphine biosynthesis pathway. This study highlights the critical role of macerozyme enzymatic pretreatment in overcoming cell wall barriers, enabling effective VIGS applications in somatic suspension cultures. The combination of VIGS and enzymatic pretreatment provides a robust platform for targeted metabolic engineering, offering insights into the regulation of morphine biosynthesis and paving the way for advancements in pharmaceutical alkaloid production and functional genomics in medicinal plants.

## 1 Introduction

*Papaver somniferum* L. (opium poppy) is an annual herbaceous plant of the Papaveraceae family, belonging to the *Papaver* genus. It has watery or milky capsules that leak when all plant parts are cut except the seeds (Tetenyi 1997). Opium poppy has been cultivated for 5000 years for food and medical use (Taşlıgil and Şahin 2018). Poppy capsules and capsule shells contain highly valuable benzylisoquinoline alkaloids (BIAs), such as noscapine, buprenorphine, morphine, papaverine, thebaine, and codeine, which are important commercial raw materials for the manufacture of semisynthetic drugs, with effects ranging from pain and spasm relief, anticancer, cough suppression, euphoria, and drowsiness (Wu and Chappell 2008).

The biochemistry and physiology of BIA metabolism in opium poppy have been studied for a long time. BIA biosynthesis begins with the L‐tyrosine transferase and consists of two branches: S‐norcoclaurine and dopamine. S‐norcoclaurin is methyl transferred to S‐reticulin by 6‐O‐methyltransferase (*N6OMT*) and 3′‐hydroxy‐N‐methyl‐(S)‐coclaurin 4′‐O‐methyltransferase (*4OMT*). On the other hand, dopamine is converted to S‐norreticulin by 4OMT and other enzymes. Then, coclaurine N‐methyltransferase (*CNMT*) converts S‐norreticulin to S‐reticulin, the starting product of the morphine synthesis pathway. In addition, (R,S)‐reticuline7‐O‐methyltransferase (*N7OMT*) and tetrahydroprotoberberine oxidase (*STOX*) catalyze the conversion of S‐norreticulin to papaverine. In one branch, reticuline oxidase (*DIOX2*) initiates a series of reactions from (R)‐reticuline to sanguinarine. In the other branch, (R)‐reticuline is converted to S‐reticuline by 1,2‐dehydroreticuline synthase (*STORR*). The conversion of S‐reticulin to thebaine is catalyzed by salutaridine synthase (*SalS*), salutaridine reductase (*SalR*), salutaridinol‐7‐O‐acetyltransferase *(SALAT*), and thebaine 6‐O‐demethylase (*T6ODM*). Codeinone reductase (*COR*) codeine O‐demethylase (*CODM*) enzymes produce codein and finally morphine (Alagoz et al. 2016; Beaudoin and Facchini 2014; Hagel and Facchini 2010, 2013; Pienkny et al. 2009; Ziegler et al. 2006). On the other hand, there are also parts of BIA biosynthesis and regulation that are not yet fully understood (Alagoz et al. 2016). The biosynthesis of alkaloids in the poppy plant is a tissue‐specific system, which is regulated by developmental and/or environmental factors (Pathak et al. 2012).

Chemical and synthetic production methods for any of the morphinan subclasses are not yet commercially viable, so opium poppy is still the only source (Guo et al. 2018). For this reason, opium poppy is important economically and industrially. Studies aimed at obtaining the desired alkaloid amounts or supporting the conversion of the unwanted product to the desired product by silencing the undesired product through genetic modifications and metabolic interventions in the pathways of poppy isoquinoline and especially morphine biosynthesis have been popular since the 2000s. (Allen et al. 2004; Davoudnia, Ahmadi, and Fabriki‐Ourang 2016; Sohrabi, Ismaili, and Nazarian‐Firouzabadi 2018; Wijekoon and Facchini 2012).

Studies on manipulation of alkaloid production in the poppy plant have focused on the *COR* (Allen et al. 2004), *SALAT* (Allen et al. 2008)*, SalR* (Kempe et al. 2009), *T6ODM*, *CODM* (Hagel and Facchini 2010; Wijekoon and Facchini 2012), and *4OMT2* (Alagoz et al. 2016) genes, which are critical in morphine biosynthesis. Although there are studies in which these gene products are silenced separately, there are also some silencing studies to improve the efficacy of the silencing (Sohrabi, Ismaili, and Nazarian‐Firouzabadi 2018).

Viruses can be designed to silence a host gene or genes of interest (Burch‐Smith et al. 2004). Hileman and his colleagues (Hileman et al. 2005) conducted a study on transferring the endogenous phytoene desaturase (PDS) gene to the tobacco rattle virus (TRV) based on this idea. The study suggested the virus‐induced gene silencing (VIGS) method as a rapid and alternative strategy for functional genomics studies on the BIA pathway genes and enzymes (Hileman et al. 2005). VIGS involves the induction of post‐transcriptional gene silencing (PTGS), a nucleotide sequence‐specific defense mechanism that targets both viral and cellular mRNAs in plants using a viral vector to generate single‐stranded RNA. Although VIGS has been used to analyze the function of specific genes in various plant species, it has been shown as an effective tool to reduce transcript levels of specific alkaloid synthase genes to increase or decrease the alkaloid levels (Wijekoon and Facchini 2012). At this point, the analysis of the target gene's cDNA is crucial in terms of silencing efficiency. Intron regions of genes do not guide VIGS, so only exons should be used for silencing a single and specific gene, the specific regions of the gene to be cloned into VIGS vectors should be scanned, and the sequence should be selected accordingly. Another factor that affects success at this stage is the choice of viral vectors depending on the target plant and tissue (Shi et al. 2021). In VIGS applications, TRV is primarily used because of its wide host range and ability to infect meristems and it causes mild symptoms, along with other commonly used vectors such as barley stripe mosaic virus (BMSV) and potato virus X (PVX) (Sung, Lin, and Chen 2014). Another important step is the method used for bacterial inoculation in Agrobacterium‐mediated gene transfer in *Agrobacterium tumefaciens* . Techniques such as needleless syringe inoculation (Senthil‐Kumar and Mysore 2014), vacuum infiltration (Tague and Mantis 2006), spraying (Singh and Kumar 2022), and wounding of plant tissues (Senthil‐Kumar and Mysore 2011) are previously reported. Besides these, various methodologies have been developed, among which enzymatic aid for genetic transformation is considered a breakthrough. One example is the utilization of maceration enzymes during the transformation. Macerozyme, a cell wall‐degrading enzyme of fungal origin, is produced mainly for protoplast isolation in plant tissue cultures. This enzyme breaks down cell wall components, including cellulose and pectin, allowing protoplasts to be released (Ramesh, Vigneshwari, and Rajeshkumar 2023). Similarly, some other studies have determined that the treatment with macerating enzymes improved Agrobacterium‐mediated transformation efficiency in plant cultures (Du et al. 2019; Kumar et al. 2006; Weber et al. 2003). These studies represent the capability of the enzyme to allow the entry of foreign DNA into plant cells by increasing the cell wall permeability, and after digestion, the area where Agrobacterium can bind to plant cells may increase. In addition, the breakdown or weakening of the cell wall may result in the release of chemicals capable of activating bacterial viral genes, hence increasing the efficiency of genetic transformation (Gelvin 2014; Pitzschke 2013; Zaltsman et al. 2010). On the other hand, the VIGS technique cannot be applied with the same efficiency in every plant species, and in some species, it is not practical because of the absence of suitable VIGS vectors. Therefore, the *A. tumefaciens* strain to be used in VIGS applications should be selected according to the target plant. Some of the *A. tumefaciens* strains used include GV3010, GV2260, AGL‐1, LBA4404, and EHA105 (Hwang, Yu, and Lai 2017). Furthermore, although Agrobacterium‐mediated transfer of VIGS vectors is possible for most plants, an effective transformation method is still a popular research topic for many plants (Orbovic and Prieto 2023; Wan et al. 2023).

In this study, specific primers were designed for different regions of the *T6ODM*, *CODM*, and *DIOX2* genes, recognized as key enzymes in morphine biosynthesis in the literature. Using these primers, the specific regions obtained were cloned into the TRV2 plasmid. To enhance silencing efficiency, unlike what has been reported in the literature (Sohrabi, Ismaili, and Nazarian‐Firouzabadi 2018), simultaneous silencing was performed by Agrobacterium‐mediated transformation of two separate vectors targeting different genes at the same time. At this stage, a novel transformation protocol was utilized, which included pretreatment of explants with the macerozyme enzyme. The expression levels of BIA genes were specified to demonstrate the successful implementation of gene silencing using the VIGS vectors designed, as well as the application of the new enzyme. With these results, a new interpretation is offered while supporting previous studies on BIA production.

## 2 Materials and Methods

### 2.1 Plant Material and Somatic Embryo Production

**FIGURE 1:**
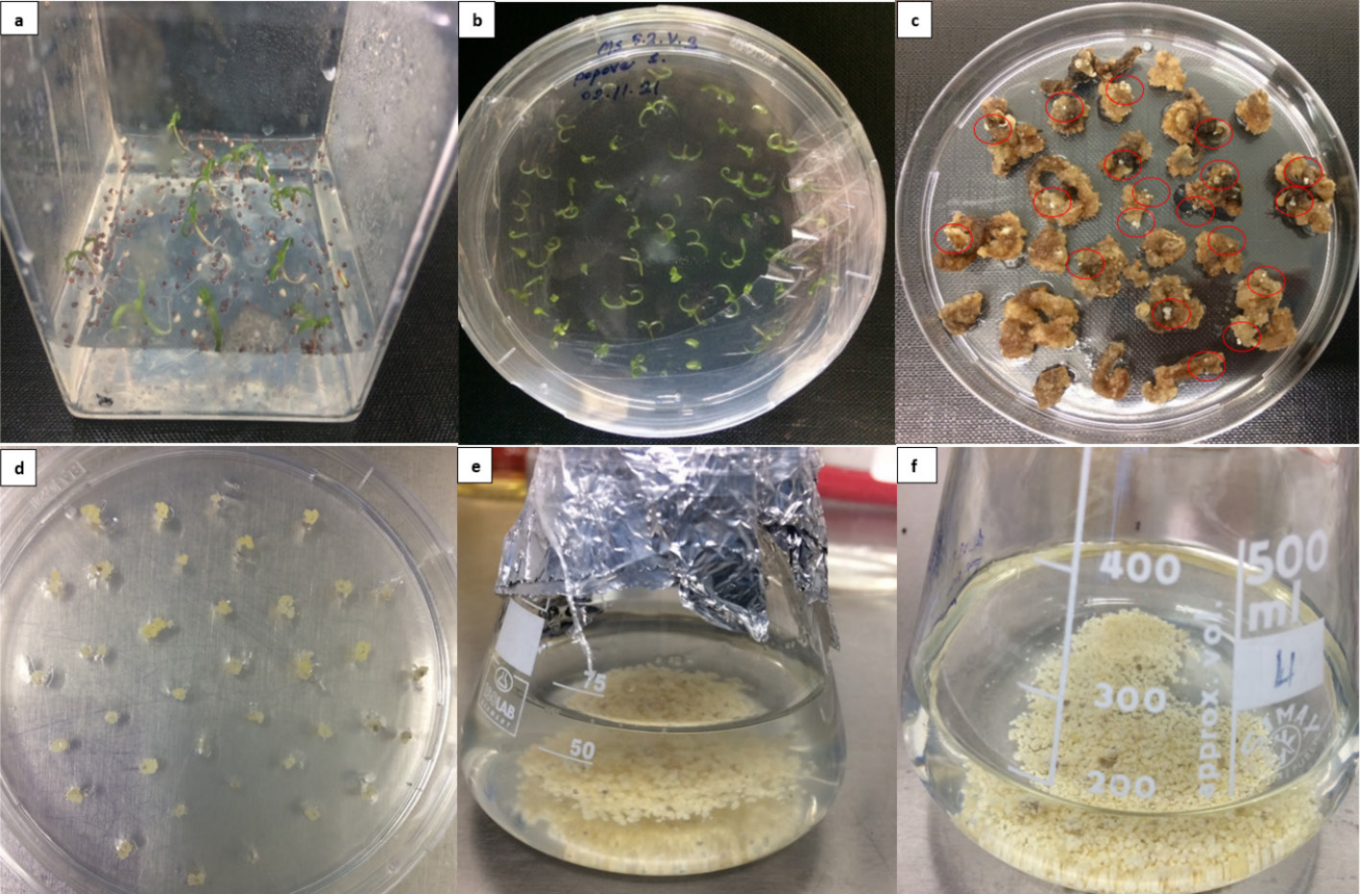
Somatic embryo induction and suspension culture steps. (a) Aseptic opium poppy sprouts, (b) explants cultured in MS‐I medium, (c) white‐round early somatic embryos on explants after 6–8 weeks, (d) globular‐shaped embryoids collected from calli, and (e–f) suspension cultures in various volumes.

In this study, seeds of *P. somniferum* belonging to the Ofis 1 variety, provided by the Turkish Grain Board (TMO), were used. The seeds were subjected to surface sterilization by soaking them in 70% EtOH for 1 min, followed by a 10‐min agitation with 0.1% Tween‐20 and 25% commercial bleach. Following surface sterilization, the seeds were rinsed with sterile distilled water and air‐dried on sterile filter paper. Planting was carried out in Magenta containers containing a hormone‐free germination medium (half‐strength MS salt and B5 vitamin, 10‐g/L sucrose, 0.5‐g/L MES, and 4‐g/L gelrite, pH: 5.8), with an average of 50 seeds per container. The planted seeds were incubated at a constant temperature of 25°C for the initial 3–4 days in the dark, followed by incubation in climate chambers with a 16:8 photoperiod until the development of seedlings (Figure 1a).

The hypocotyl‐cotyledon explants obtained from 1‐week‐old aseptic seedlings were utilized as the explant source (Figure 1b). The explants were cultured in MS‐I medium (full‐strength MS salts and B5 vitamin, 1.75‐mg/L NAA, 0.125‐mg/L kinetin, 30‐mg/L ascorbic acid, 15‐g/L sucrose, 4‐g/L gelrite, and 1‐mL/L PPM) for somatic embryo induction. The cultures were maintained in the dark at 21°C in a growth chamber and subcultured every 10 days in the same medium. After 6–8 weeks, globular‐shaped embryoids were collected on the calli (Figure 1c) and initially transferred to solid MS‐S medium (full‐strength MS salts and B5 vitamin, 15‐g/L sucrose, 1‐mL/L PPM, 45‐mg/mL ascorbic acid, pH: 5.8) and cultured for 1 week (Figure 1d). Then, they were transferred to Erlenmeyer flasks containing liquid MS‐S medium for the development of embryogenic suspension culture. Somatic embryos were subcultured into fresh MS‐S medium every week with a 1:1 increase in volume (Figure 1e–f).

### 2.2 Designing Inserts and Building pTRV2 Vectors for VIGS

It is known that the *CODM* (Acc: GQ500141), *T6ODM* (Acc: GQ500139), and *DIOX2* (Acc: GQ500140) genes selected as targets exhibit a high degree of sequence similarity (Li et al. 2020). For this purpose, genomic sequence information for each entry was examined to select critical regions in the designs. A phylogenetic map was created using ClustalOmega tool (https://www.ebi.ac.uk/jdispatcher/msa/clustalo). Percentage similarity matrices for the genes subjected to multiple sequence alignment were calculated. This facilitated the selection of specific regions for the target genes in primer designs.

For the generation of VIGS inserts, suspension cultures of the Ofis1 variety were used as RNA source. Total RNA isolation from 20 mg of freeze‐dried cells was carried out using the Direct‐zol RNA Kit (Zymo) according to the kit protocols. Subsequently, 1 μg of total RNA was reverse transcribed into cDNA using the RevertAid First Strand cDNA Synthesis Kit (Thermo Scientific) and oligo (dT) primers. Primers were designed to incorporate BamHI and *Xho*I restriction sites at the 5′ and 3′ ends, respectively, of the final product oligos, based on the *CODM*, *T6ODM*, and *DIOX2* sequences (Table S1a), to be used in creating inserts for VIGS vectors. Amplicons for the *CODM*, *T6ODM*, and *DIOX* regions were generated using the synthesized cDNA and designed primers.

The resulting amplicons and the pTRV2 (Addgene #148968) vector were double digested with *BamH*I and *Xho*I restriction endonucleases, and the sticky ends were ligated using T4 DNA ligase enzyme. The ligated products were then cloned into the *E. coli* DH5α strain, and colonies verified by PCR were subjected to plasmid isolation. The isolated plasmids were transferred to the *A. tumefaciens* LBA4404 competent strain via 2400 voltage, 2‐mm cuvette size electroporation. Simultaneously, the pTRV1 (Addgene #148969) vector, in its original form, was transferred to the LBA4404 strain. All bacteria cultures were kept in −86°C freezers as glycerol stocks for long‐term storage.

All created vectors are labeled as shown in Table 1.

**TABLE 1:** Final vectors were labeled as listed.

Target gene	Vector name (will be labeled without pTRV2 in the further text)
*CODM*	V2‐pTRV2
*CODM*	V3‐pTRV2
*T6ODM*	T1‐pTRV2
*T6ODM*	T2‐pTRV2
*DIOX2*	D‐pTRV2

The list of primers used for cloning and verification is provided in Table S1b.

### 2.3 *A. tumefaciens* –Mediated Temporary Gene Silencing in Somatic Embryos With Weakened Cell Walls Using Macerozyme

For genetic manipulation studies, somatic embryos were subjected to transient gene silencing experiments using various combinations of *Agrobacterium* strains produced within the scope of the study.

One night before transformation, all *Agrobacterium* strains (pTRV1, pTRV2, and pTRV2 + VIGS inserts) were grown in a 50‐mL liquid LB medium containing appropriate antibiotics at 28°C, with shaking at 200 rpm, for 16–18 h. At the end of this period, the bacterial cultures were centrifuged to pellet the bacteria, and the existing growth medium was removed. The bacterial pellet was resuspended in 10 mL of MS0 medium (full‐strength MS salts and B5 vitamins, 20‐g/L sucrose, pH: 5.8) supplemented with 100‐μM acetosyringone and 0.1 M D‐mannitol.

The embryos intended for transient gene silencing were aseptically removed from their culture medium and washed once with MS0 medium. Then, the washing medium was discarded, and MS‐E medium (full‐strength MS salts and B5 vitamins, 20‐g/L sucrose, 0.25 M D‐mannitol, 10‐mM CaCl_2_, 0.01 M MES, pH: 5.8) containing 0.15% macerozyme enzyme (filter sterilized) was added. The embryos were incubated in an orbital shaker at 30°C, 135 rpm, in the dark for 5 min. At the end of this period, the embryos were washed twice with a wash medium containing MS0 + 0.25 M mannitol to remove the enzyme medium. To assess the effectiveness of the enzyme application, the same experiment was conducted without the inclusion of macerozyme.

To achieve transient gene silencing, the enzyme‐treated embryonic cell culture was incubated in the dark at 23°C, with shaking at 130 rpm for 5 h, with pTRV1‐LBA4404 and pTRV2‐VIGS‐LGA4404 (Table 2) added to the culture, with each having a final OD600 value in the range of 0.1–0.2. After the incubation period, the cells were washed twice with ice‐cold distilled water and frozen in liquid nitrogen. The frozen cells were lyophilized in a freeze drier. The dried cells were aliquoted and stored in a −86°C deep freezer until RNA isolation.

**TABLE 2:** Transformation combinations. To improve the comprehensibility of transformation combinations within the manuscript, the abbreviations provided in the “Label” column of the table have been consistently used throughout the Results and Discussion sections. In the subsequent text, applications referred to as “Label” indicate standard VIGS treatments, whereas those denoted as “Label *+* Enz” refer to VIGS treatments preceded by macerozyme pretreatment.

Target gene	Vector	Label	Target gene	Vector	Label
*CODM*	V2 + pTRV1	V2	*CODM + DIOX2*	V2 + D + pTRV1	V2D
*CODM*	V3 + pTRV1	V3	*CODM + DIOX2*	V3 + D + pTRV1	V3D
*T6ODM*	T1 + pTRV1	T1	*T6ODM + DIOX2*	T1 + D + pTRV1	T1D
*T6ODM*	T2 + pTRV1	T2	*CODM + T6ODM*	V3 + T1 + pTRV1	V3T1
*DIOX2*	D + pTRV1	D	Control	pTRV2 + pTRV1	Control

### 2.4 Quantitative Reverse Transcription PCR

Freeze‐dried cells were crushed in liquid nitrogen, and approximately 30 mg of grounded cells were used per sample for total RNA isolation. Extraction was performed using a column‐based Direct‐zol RNA Kit (Zymo). cDNA was synthesized from 1500 ng of DNaseI‐treated total RNA using oligodT primers and RevertAid First Strand cDNA Synthesis Kit (Thermo Scientific).

Primers were designed for the coding regions of the enzymes CODM, T6ODM, DIOX2, and COR that were used to investigate changes in the expression of these genes (Table S2). Quantitative PCR reactions were conducted in a 20‐μL volume, with each primer at a concentration of 0.4 μM, using SsoAdvanced Universal SYBR Green Supermix (BioRAD). Biological samples of each application were bulked together, and three technical replicates were set up. The actin housekeeping gene was used for normalization. Comparative gene expression analyses were performed using the 2^−ΔΔCt^ method (Livak and Schmittgen 2001).

## 3 Results

### 3.1 Developing Somatic Embryo Cultures

The success of the production method described in this report for obtaining somatic embryos derived from hypocotyl‐cotyledon explants has been determined. After a period of 6–8 weeks following the initial acquisition of the first somatic cells and the initiation of the suspension culture, it was observed that the cell quantity consistently and rapidly increased (Figure 1).

### 3.2 Design of VIGS Plasmid

**FIGURE 2:**
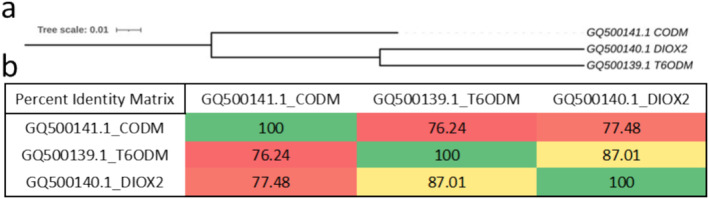
The phylogenetic relationship of *CODM*, *T6ODM*, and *DIOX2* genes derived from *P. somniferum* . (a) Alignment and phylogenetic tree were generated using ClustalOmega with the genomic DNA information of the relevant genes. (b) The percentage similarity matrix shares the percentage results of the similarity between *CODM*, *T6ODM*, and *DIOX2* genes obtained through multiple sequence alignment.

The genomic DNA sequences, specified with accession numbers in Figure 2a,b, were obtained from the NCBI database, and a comparative analysis was performed. *T6ODM* and *DIOX2* genes exhibit approximately 87% similarity to each other. The *CODM* gene shows a similarity of ~77% to these other two genes.

**FIGURE 3:**
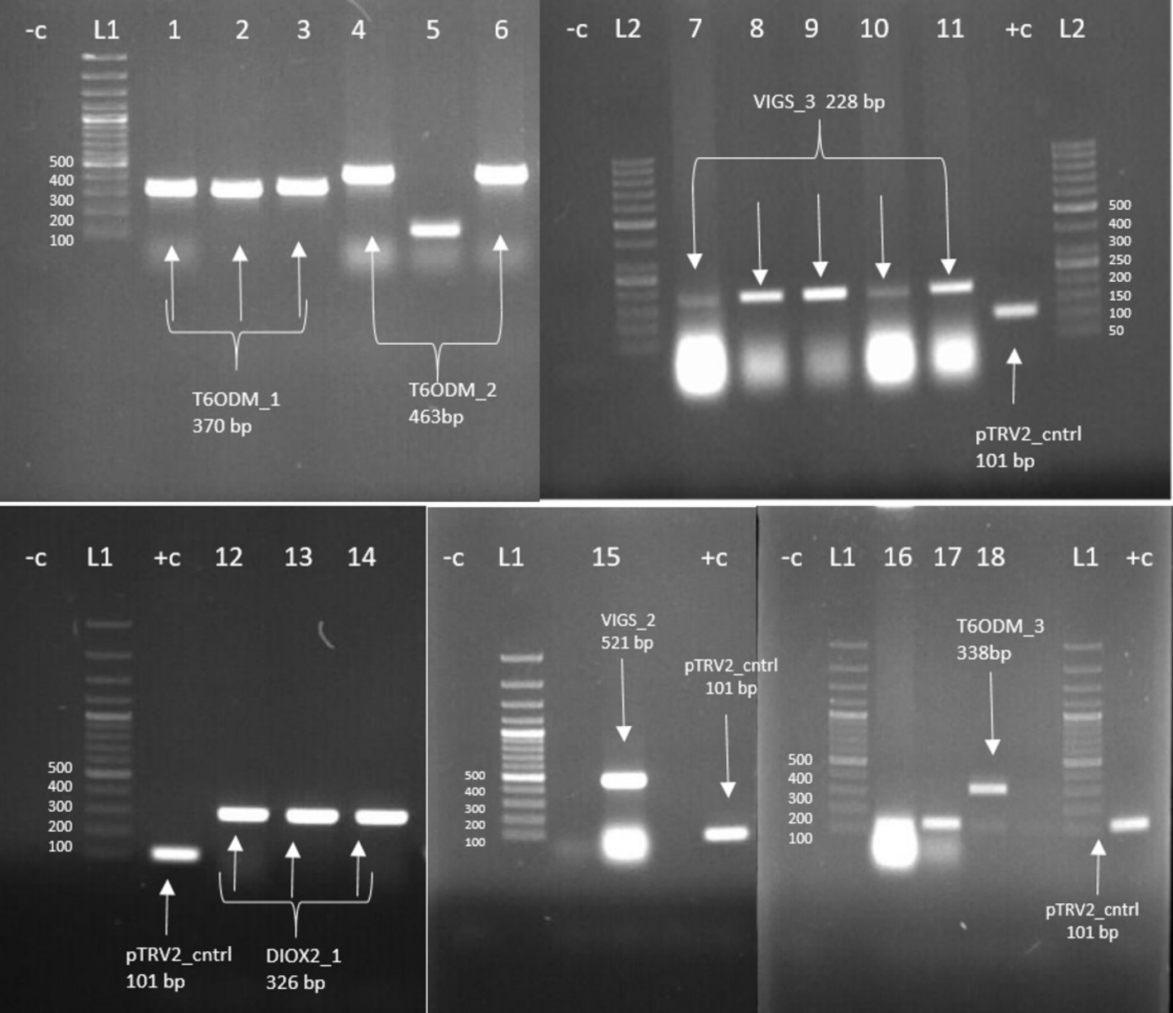
The colony PCR analysis gel images of VIGS‐pTRV2 plasmids in *E. coli* DH5α. Ladder: SM0373 (Thermo Scientific).

The VIGS inserts were generated from the RNA of somatic embryos, where they were sourced, using the primers listed in Table S1a. After restriction and ligation reactions, they were transferred to *E. coli* DH5α strains. Colonies were screened based on previously calculated product size differences using the TRV2_CONT primer listed in Table S1b (Figure 3). The products confirmed by PCR were used in genetic manipulation experiments.

### 3.3 Gene Expression Analysis in BIA Pathway and Effects of Macerozyme Treatment

**FIGURE 4:**
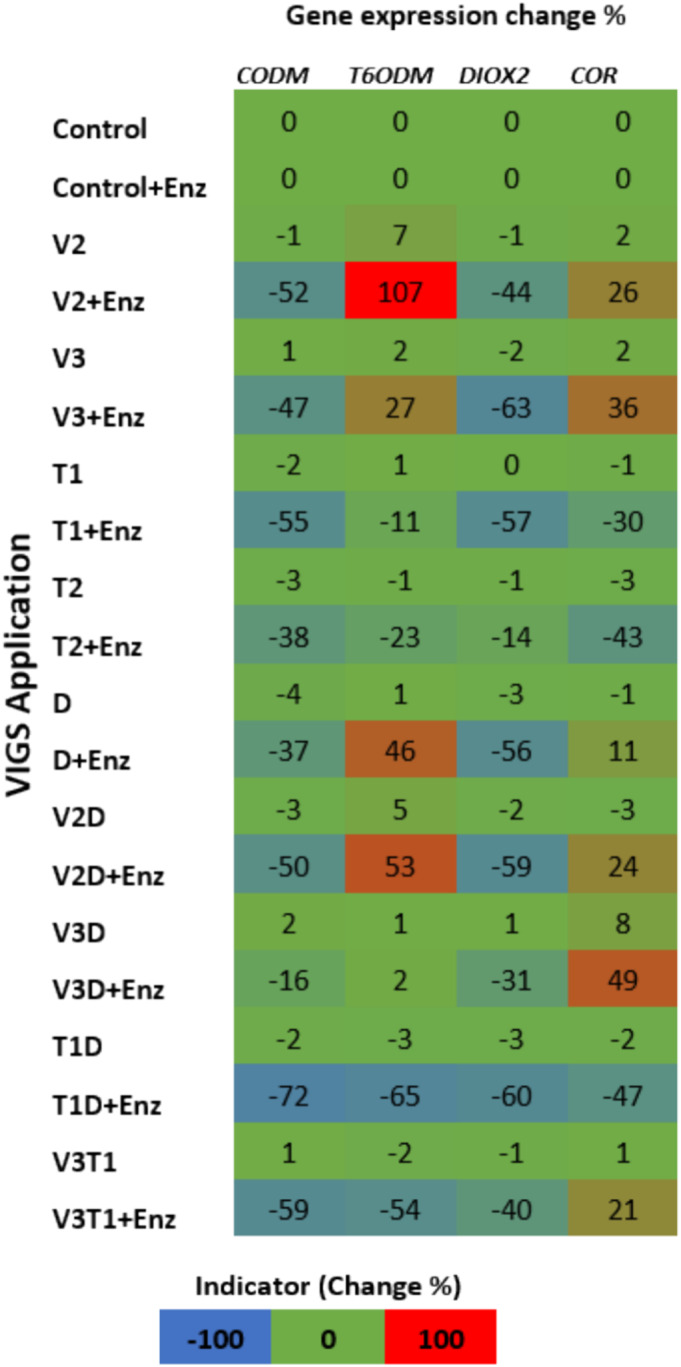
Changes in gene expression compared to control are presented as percentages in the heat map (created with Microsoft Excel).

The VIGS application was conducted in a total of 20 treatments, including a control, with a difference in 10 genetic applications, either containing or not containing macerozyme. In the transformation experiments without macerozyme pretreatment, no change in gene expression was observed. In experiments with macerozyme pretreatment, increases and decreases were observed in the expression profiles of *CODM*, *T6ODM*, *DIOX2*, and *COR* genes compared with the control. All results were submitted as heatmap image in Figure 4.

The most suppressive application on *CODM* gene expression was observed to be the T1D combination, causing a 72% decrease. This rate was followed by decreases of 59% in V3T1, 55% in T1, 52% in V2, 50% in V2D, 47% in V3, 38% in T2, 37% in D, and 16% in V3D applications. No increase in *CODM* gene expression was observed in any VIGS applications.

Although the most suppressive application on *T6ODM* gene expression was observed to be the T1D combination, causing a 65% decrease, the V3T1 application also showed a 54% reduction in gene expression. However, V2, V3, D, and V2D applications demonstrated an enhancing effect on gene expression, increasing gene expression by 107%, 27%, 46%, and 53%, respectively. No change in gene expression was observed in the V3D application.

In experiments conducted on *DIOX2* gene expression, decreases in gene expression were observed in all VIGS combinations ranging between 14% and 63%. The most suppressive application on gene expression was determined to be V3 with a reduction of 63%, followed by T1D (60%), V2D (59%), T1 (57%), D (56%), V2 (44%), V3T1 (40%), V3D (31%), and T2 (14%). No increase in gene expression of the *DIOX2* gene was observed in any of the VIGS applications.

The most suppressive application on *COR* gene expression was T1D, leading to a reduction of 47%. However, in the V3D application, a 49% increase in gene expression was observed. Although T1 and T2 applications showed reductions of 43% and 30% in gene expression, increases in gene expression levels were observed in V3, V2, V2D, D, V3T1, and V3D applications, with percentages of 36%, 26%, 24%, 21%, and 11%, respectively.

## 4 Discussion

The research embarked on a new effort using the VIGS technique to effectively manipulate alkaloid production in *P. somniferum* L. The primary focus was on the selective silencing of essential genes involved in morphine biosynthesis, such as *CODM*, *T6ODM*, *COR*, and *DIOX2*. A new VIGS protocol was reported that included primer development, construction of pTRV2 vectors, and use of the macerozyme enzyme for gene transfer into somatic embryos. The success of this approach was evident in the results section, where somatic embryo cultures flourished, and VIGS effectiveness was corroborated through gene expression analysis. Particularly noteworthy was the impact of macerozyme treatment on gene transformation, underscoring the transformative potential of this introduced enzyme. Additionally, the lack of change in expression levels in samples without enzyme treatment was likely due to the absence of successful genetic manipulation. This result suggests that using the enzyme enables successful genetic manipulation.

The efficiency of the VIGS technique in reshaping gene expression related to morphine biosynthesis was underscored. A meticulous analysis of suppressive and enhancing effects on specific genes unfolded a nuanced understanding of alkaloid modulation. Notably, the exploration extended to the implications of macerozyme treatment, marking a critical evaluation of the study's outcomes.

In summary, an effective study was achieved toward the manipulation of alkaloid production in *P. somniferum* L. The integration of VIGS and the macerozyme enzyme treatment not only advanced the understanding of morphine biosynthesis pathway alterations but also offered prospective applications in the pharmaceutical and industrial realms.

Challenges were encountered during the primer design phase, emphasizing the high sequence similarity among *CODM* and *T6ODM* genes. This observation aligns with existing literature, affirming the pivotal roles of these genes in morphine biosynthesis (Guo et al. 2018; Li et al. 2020; Zhang et al. 2022). In response, a strategic approach was adopted, targeting areas with the most significant differences toward the 3′ ends of the coding sequences of these genes.

This study was conducted to investigate an alternative way of genetically interfering with the synthesis pathway in Ofis 1 variety, which is known to be rich in morphine content. The production of alkaloids in poppy suspension cultures was initially published long ago (Tam, Constabel, and Kurz 1980). It has been reported that the removal of hormones from the culture medium results in an increase in the amounts of morphine and codeine compared with hormone‐containing cell suspension cultures (Siah and Doran 1991). Therefore, culture environments were designed to be free from hormones. The introduction of VIGS plasmids via *A. tumefaciens* transformation and the subsequent pretreatments showcased the resilience of the approach. The efficacy of the method became evident in the successful direct application of gene silencing to somatic embryos. This not only streamlined the process compared with conventional techniques but also presented a favorable alternative for future permanent gene editing experiments. The enzymatic method, surpassing traditional techniques such as microprojectile and sonication‐assisted methods, demonstrated its potential in callus‐somatic embryo cultures (Chowdhury and Vasil 1992; Santos et al. 2019; Trick and Finer 1998). The unique enzymatic application method, inspired by Batth et al.'s protoplast isolation protocol, presented a breakthrough, ensuring the success of gene transformation through partial cell wall removal (Batth et al. 2021).

Delving into the outcomes of VIGS applications, a consistent decrease in the expression of *CODM* and *DIOX* genes across various combinations underlines the potency of the approach. Unexpectedly, certain VIGS applications, notably T1, T2, D, and T1D, exhibited a decrease in *CODM* gene expression, contrary to expectations, potentially attributed to sequence similarities among the targeted genes (Sohrabi, Ismaili, and Nazarian‐Firouzabadi 2018).

The pivotal *COR* gene, responsible for morphine conversion, exhibited consistent responses. The increase in *COR* gene expression in V2 and V3 applications targeting the *CODM* gene aligns with expectations. Conversely, T1 and T2 applications targeting *T6ODM* resulted in decreased *COR* gene expression, indicating the complex interplay of genes in morphine biosynthesis. Dual‐targeting applications (V2D and V3D) resulted a substantial increase in *COR* gene expression, signifying the potential for synergistic effects.

*T6ODM* gene expression exhibited a consistent decrease in applications targeting the gene directly, whereas an increase was observed in applications targeting the *CODM* gene. These findings resonate with prior literature, validating the reported declines and increases in gene expression (Wijekoon and Facchini 2012).

The study's unique contribution lies in the deliberate decrease in *CODM* gene expression, inhibiting morphine conversion, while concurrently promoting codeine synthesis through *COR* gene upregulation. Importantly, *T6ODM* gene expression remains unaffected, allowing the pathway to remain open for codeine production. The proposed design of specific target regions in *T6ODM*, *CODM*, and *DIOX2* genes sets the stage for future modifications in codeine‐morphine and thebaine alkaloids.

Beyond these alkaloids, the study unraveled the potential for secondary product modulation. Targeting the *DIOX2* pathway stimulated the papaverine pathway, resulting in papaverine production as a secondary product. Sequences targeting the *DIOX2* gene also showcased the potential for modifying the pathway's branching point, impacting sanguinarine production. These findings align with Davoudnia et al.'s study, validating the broader implications of the approach (Davoudnia, Ahmadi, and Fabriki‐Ourang 2016).

In tandem with evolving methodologies, the study deviated from the conventional use of a single vector in VIGS applications. Simultaneously transferring two separate vectors containing different gene regions through *Agrobacterium* yielded promising results. Dual‐vector applications, such as T1D and V3T1, demonstrated enhanced gene silencing effects compared to individual vector applications, showcasing the potential of the dual‐vector system for simultaneous gene modulation.

In conclusion, the study marks a pioneering effort in the manipulation of alkaloid production, offering not only a deepened understanding of morphine biosynthesis but also providing a platform for future advancements in gene editing and alkaloid engineering. The meticulous integration of VIGS, precise primer design, and innovative transformation techniques positions this work at the forefront of research in plant molecular biology, holding promise for applications in diverse fields.

## Supporting information


**Data S1.** Peer Review.

**Data S2.** Supporting Information.

**Data S3.** Supporting Information.

**Data S4.** Supporting Information.

**Data S5.** Supporting Information.

**Data S6.** Supporting Information.

**Data S7.** Supporting Information.

**Table S1.** Supporting Information.
**Table S2.** Primers used to quantify gene expression levels.
