# Supplementary material for: Modification of Gene Expression Involved in Alkaloid Production in Opium Poppy by VIGS Combined With Pretreatment of Macerozyme Enzyme
Source: Plant Direct. 2025 Jan 7;9(1):e70034. doi: 10.1002/pld3.70034 (PMC11706800; doi:10.1002/pld3.70034)

This document was created to explain in detail the original versions of the agarose gel images in the manuscript. Since there are bands of different studies in the original agarose gel images, the parts of the images belonging to this study were taken and combined by cut-and-paste method within the text, by also marking the original versions sent in the attachment.

Original gel images are named as below:

| File name | Label in manuscript image |
| --- | --- |
| geloriginal(1) | A |
| geloriginal(2) | B |
| geloriginal(3) | C |
| geloriginal(4) | D |
| geloriginal(5) | E |

**E**

**D**

**C**

**B**

**A**

*geloriginal(1)*


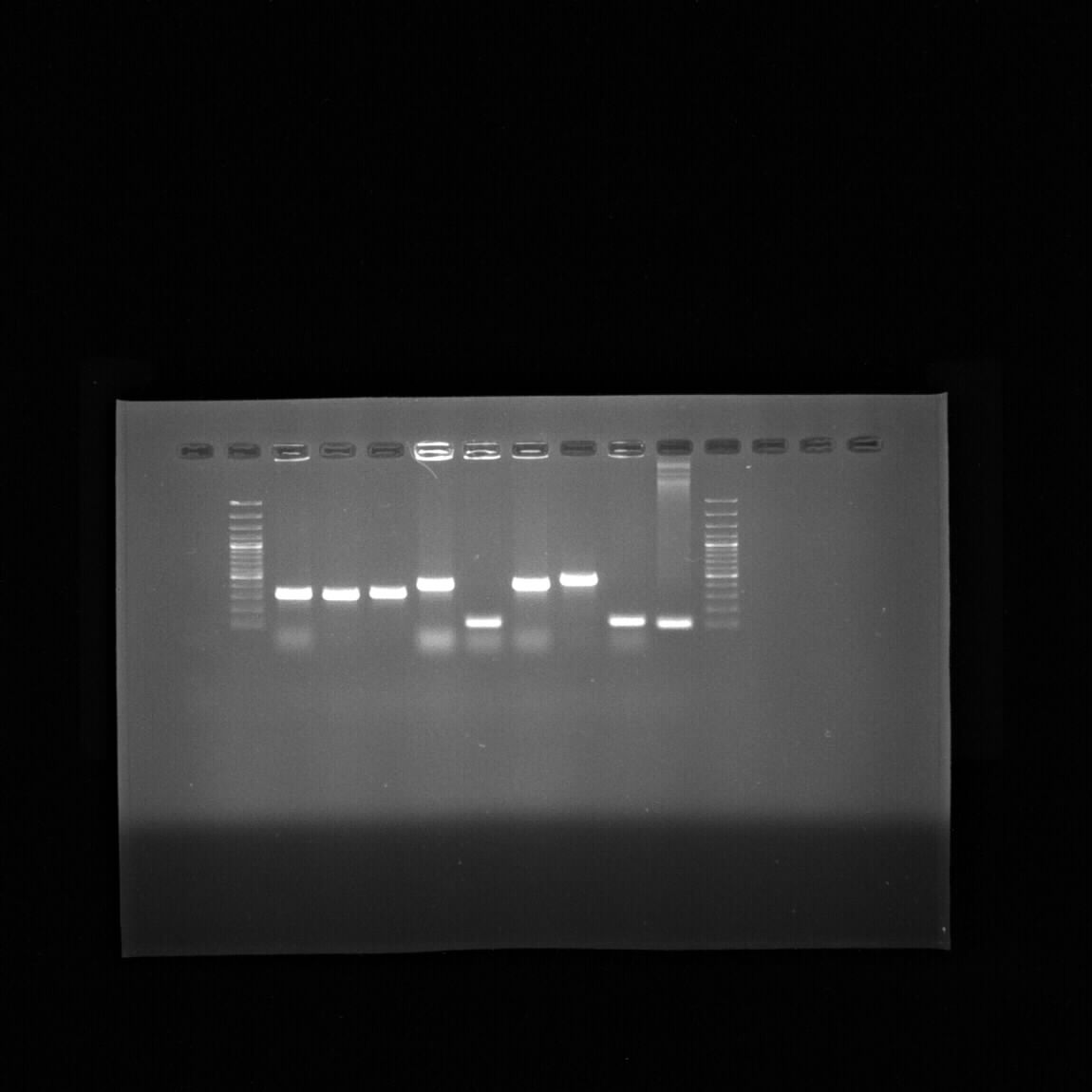


**A**

*geloriginal(2)*


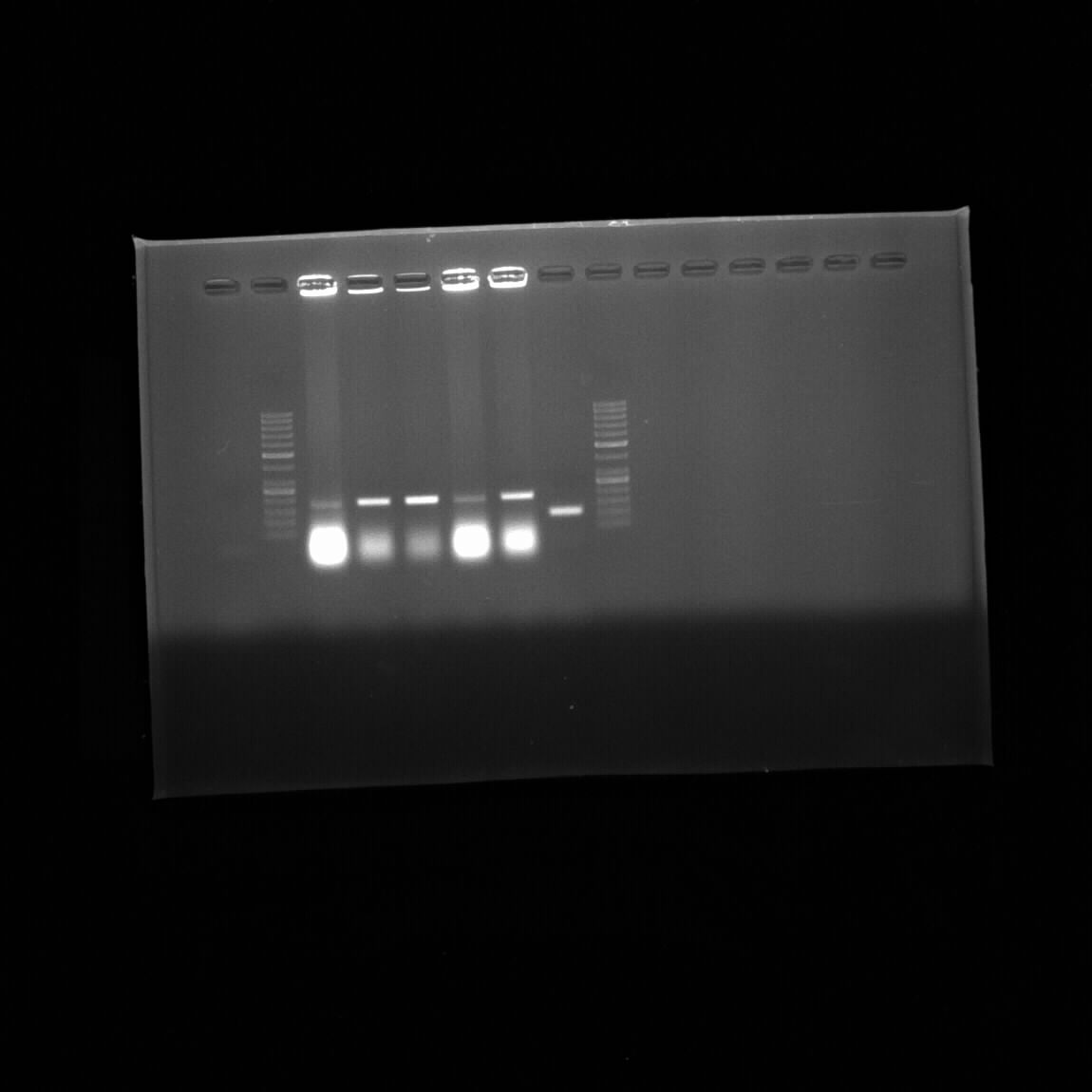


**B**

*geloriginal(3)*


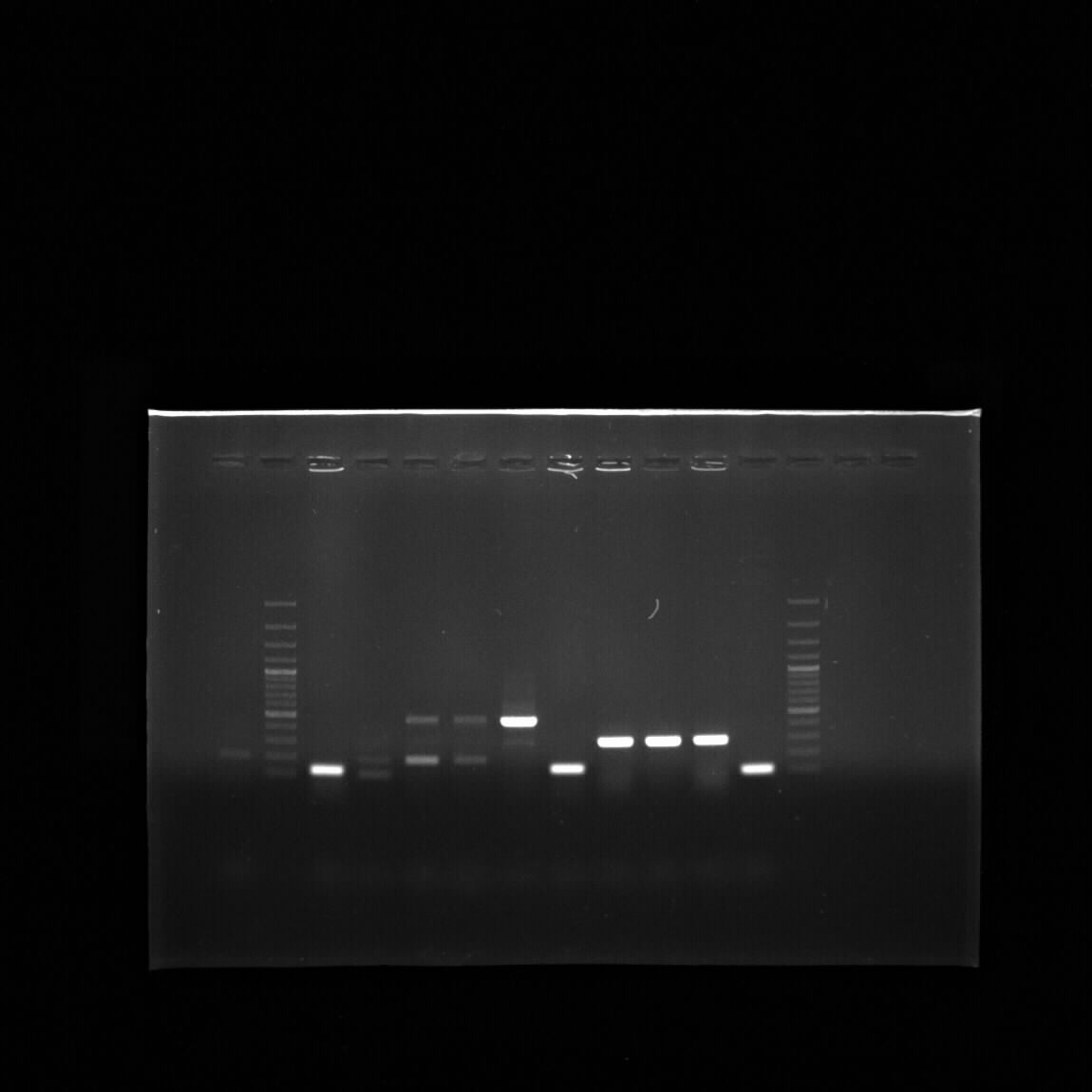


**image is mirrored in the manuscript**

**C**

*geloriginal(4)*


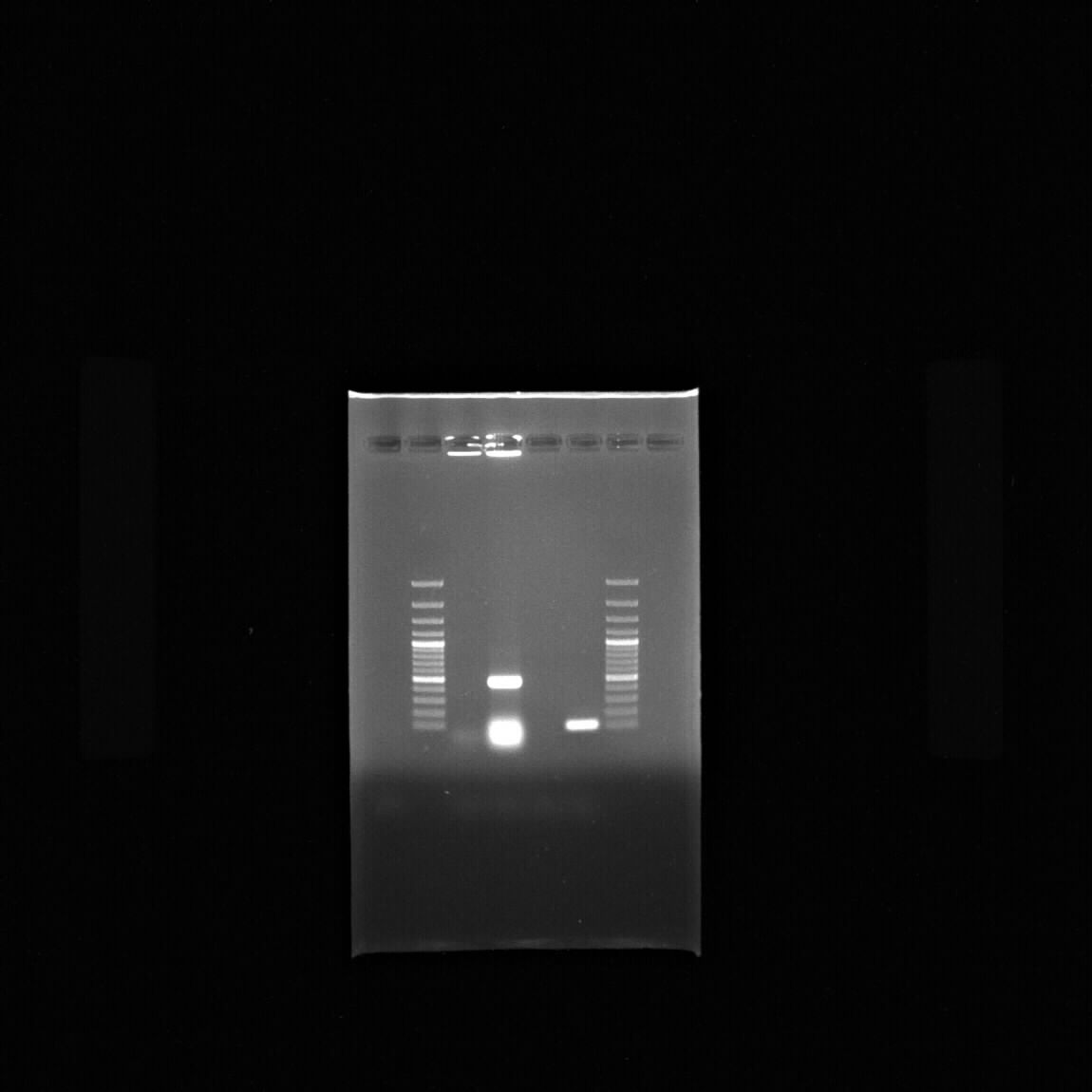


**D**

geloriginal(5)


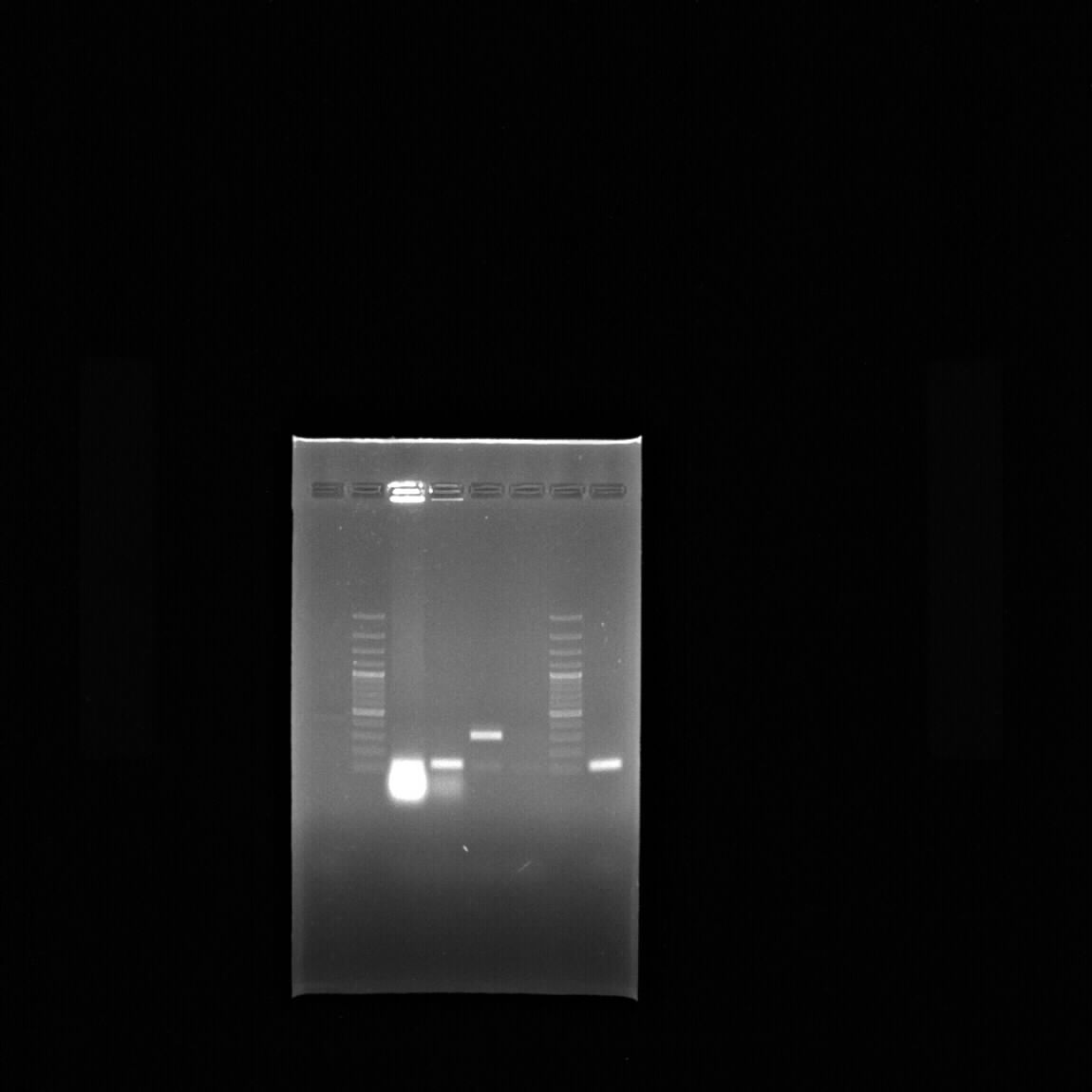


**E**


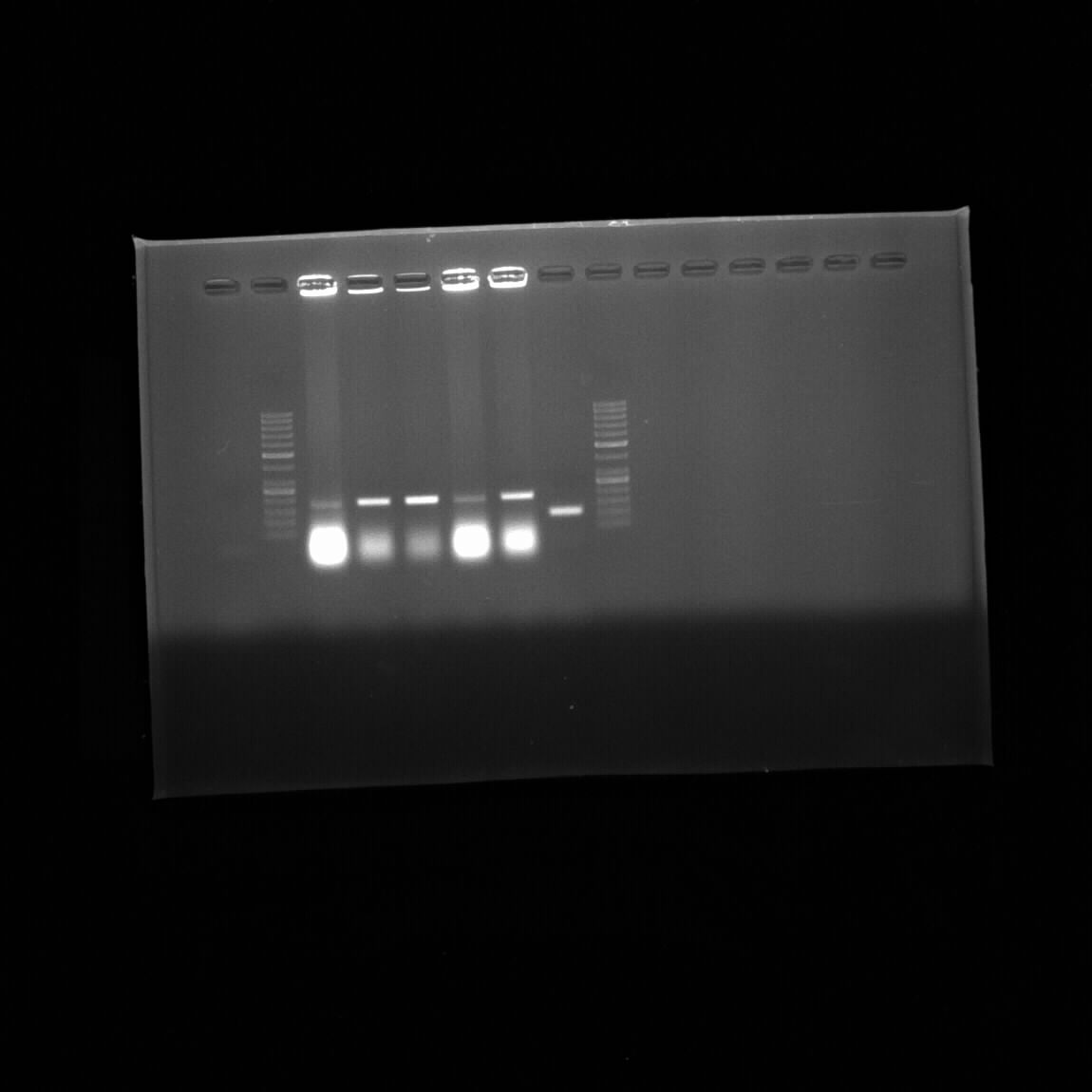

Supplement: Supplementary file 2 — Data S2. Supporting Information. [file PLD3-9-e70034-s003.docx]
