# Supplementary figures and images for: Modification of Gene Expression Involved in Alkaloid Production in Opium Poppy by VIGS Combined With Pretreatment of Macerozyme Enzyme
Source: Plant Direct. 2025 Jan 7;9(1):e70034. doi: 10.1002/pld3.70034 (PMC11706800; doi:10.1002/pld3.70034)

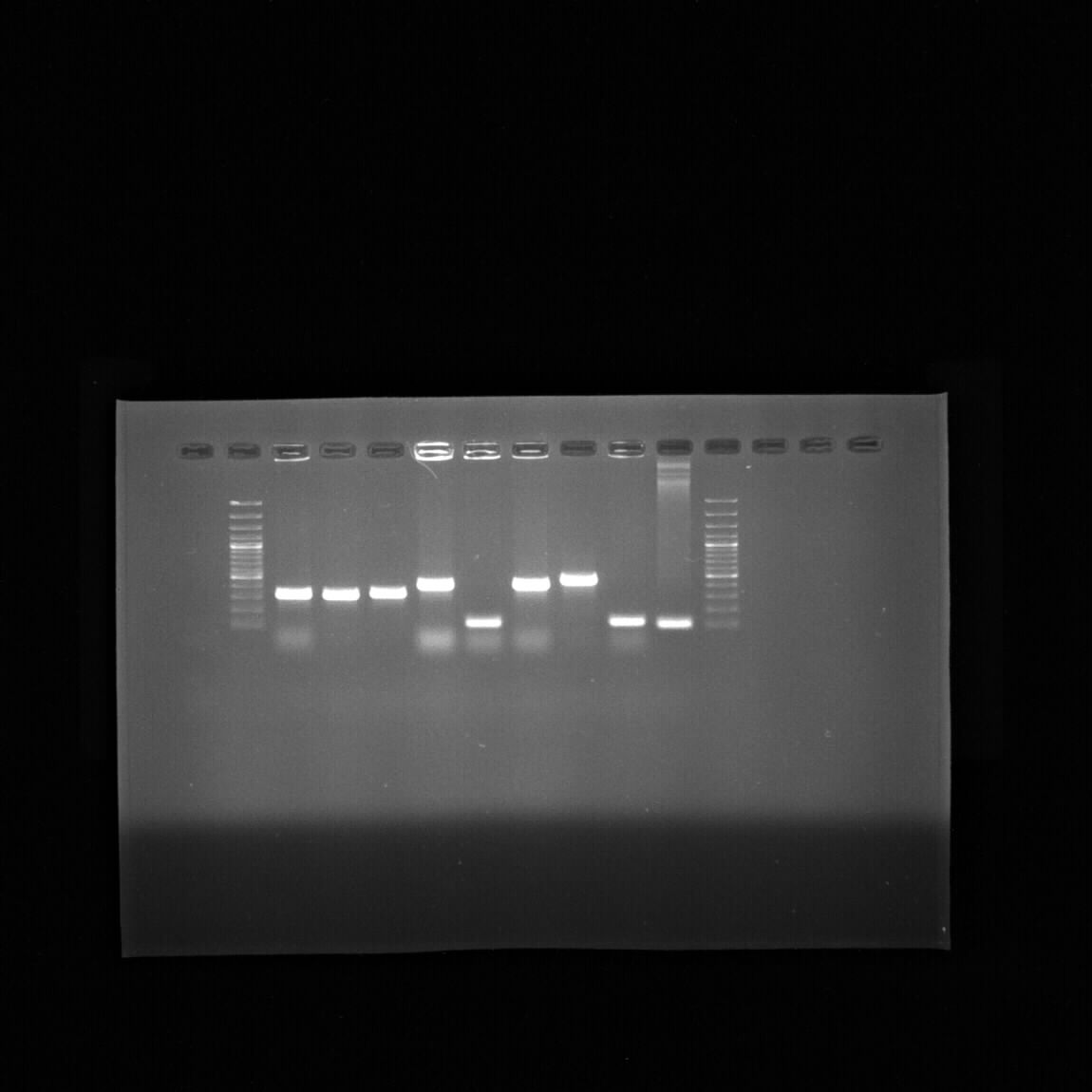

Supplement: Supplementary file 3 — Data S3. Supporting Information. [file PLD3-9-e70034-s001.jpg]

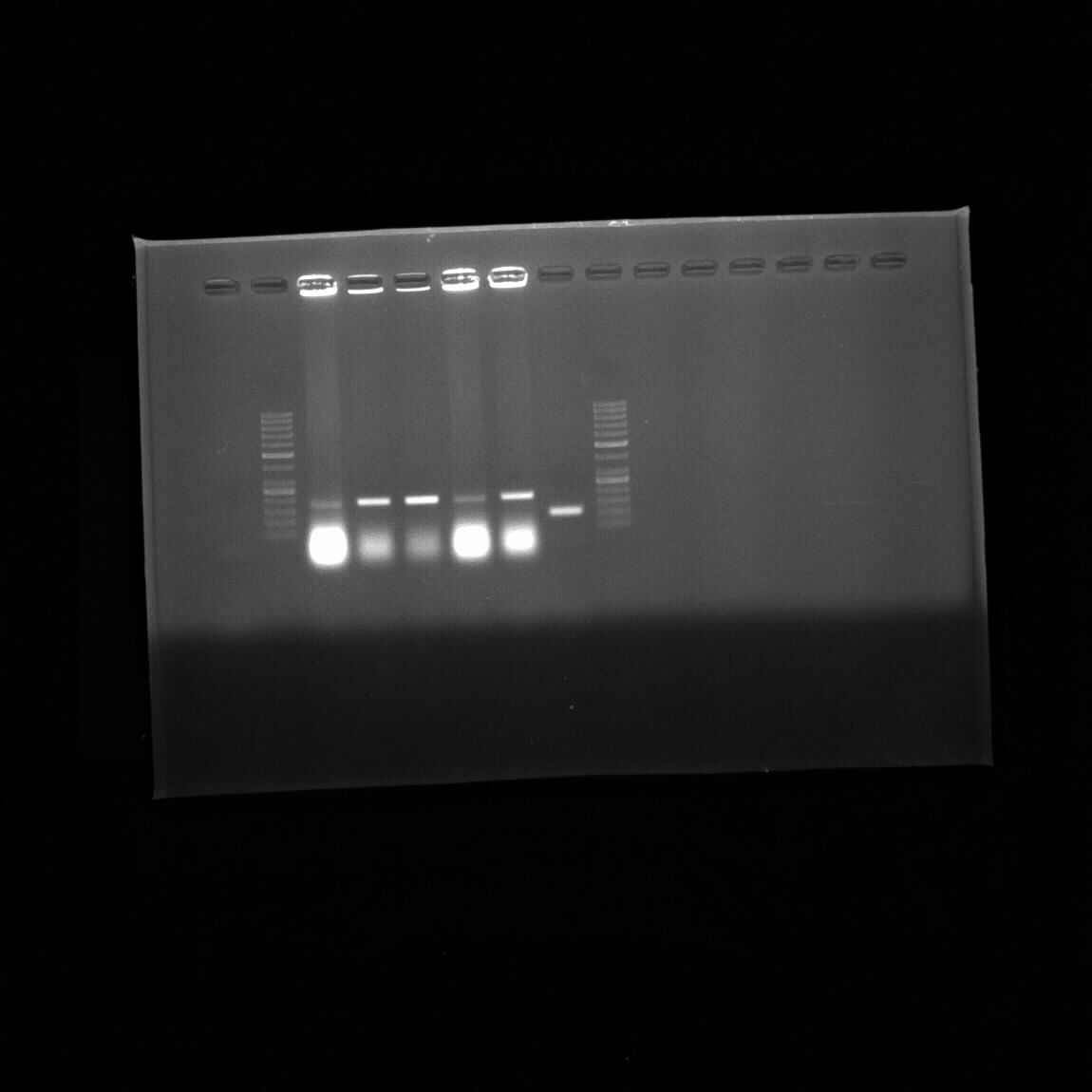

Supplement: Supplementary file 4 — Data S4. Supporting Information. [file PLD3-9-e70034-s006.jpg]

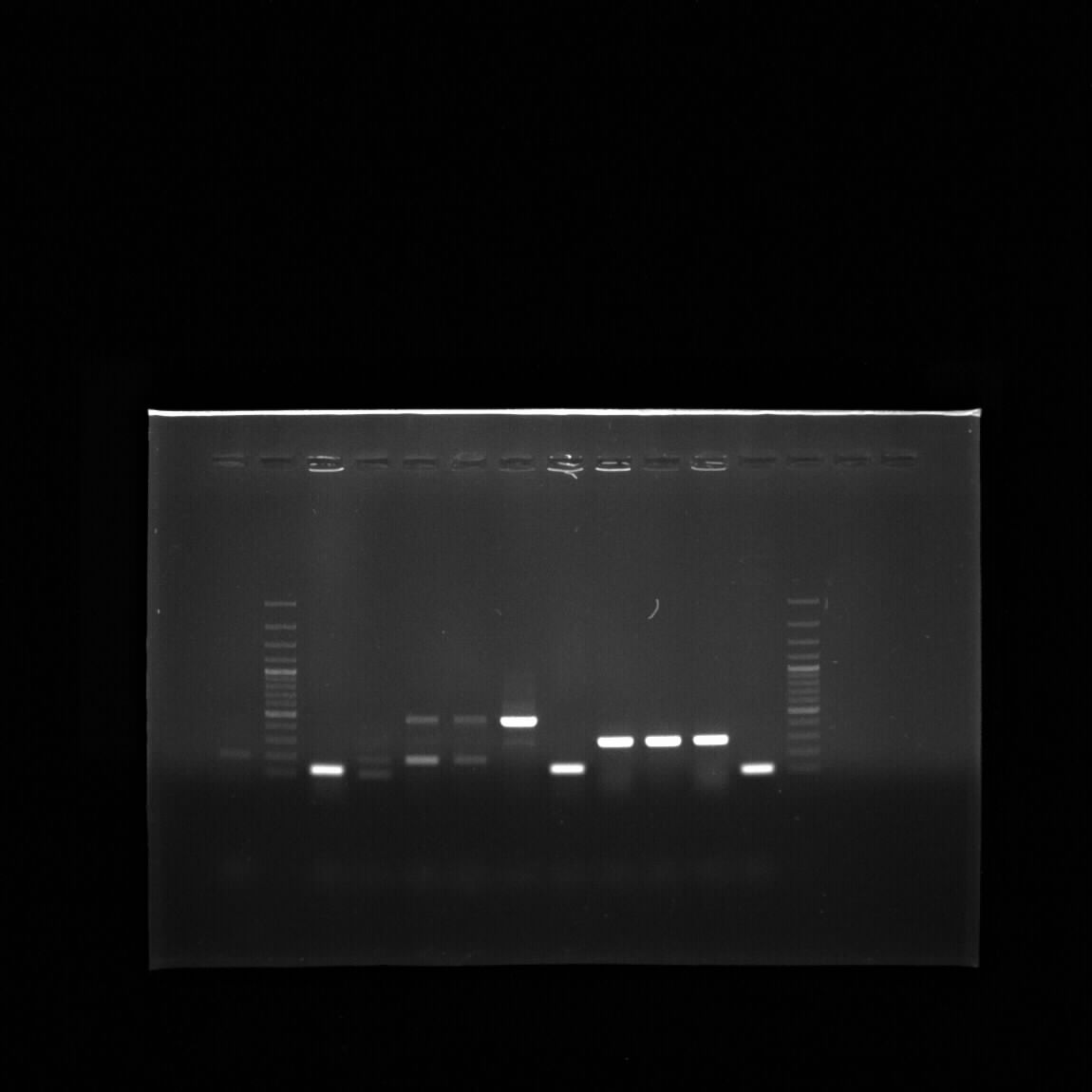

Supplement: Supplementary file 5 — Data S5. Supporting Information. [file PLD3-9-e70034-s004.jpg]

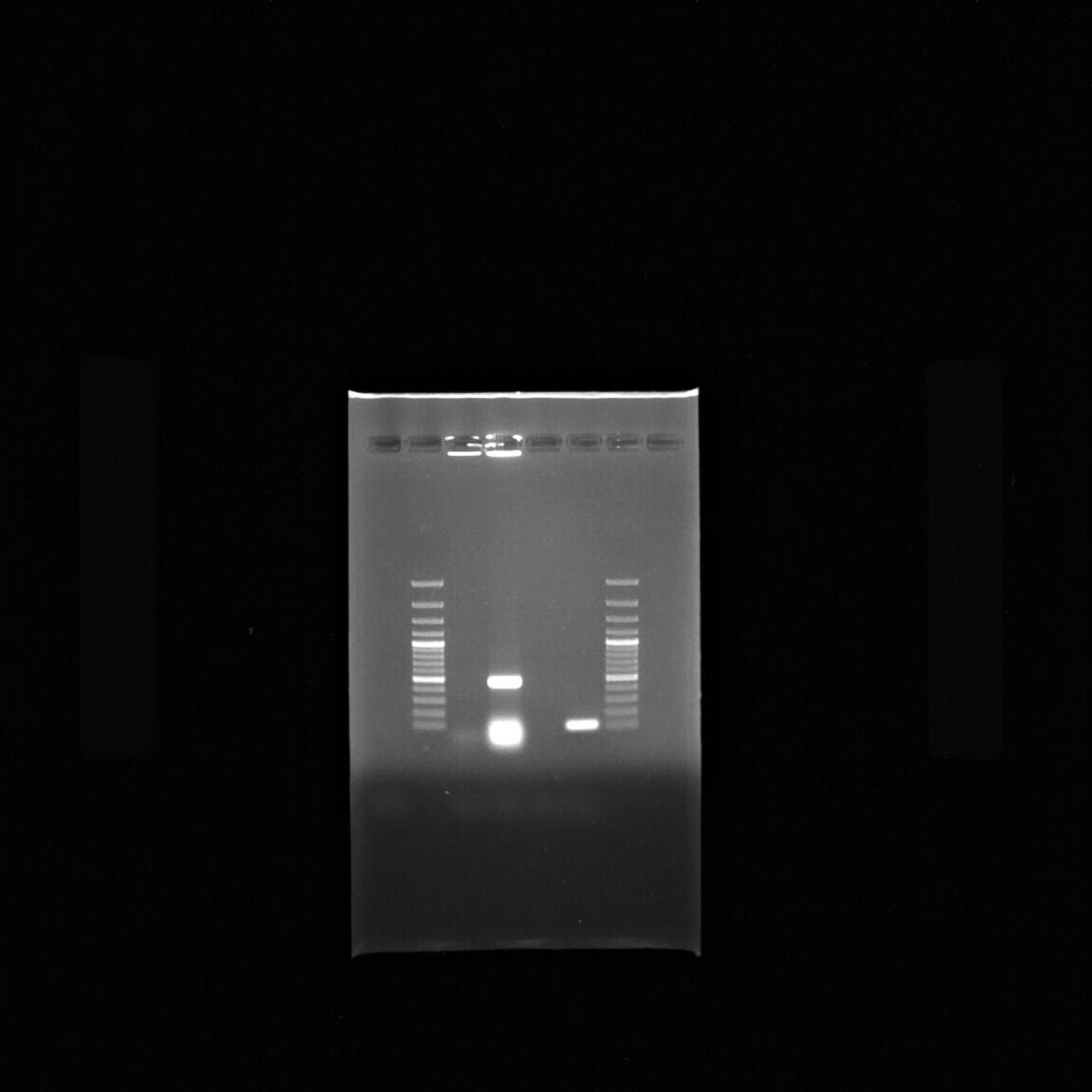

Supplement: Supplementary file 6 — Data S6. Supporting Information. [file PLD3-9-e70034-s005.jpg]

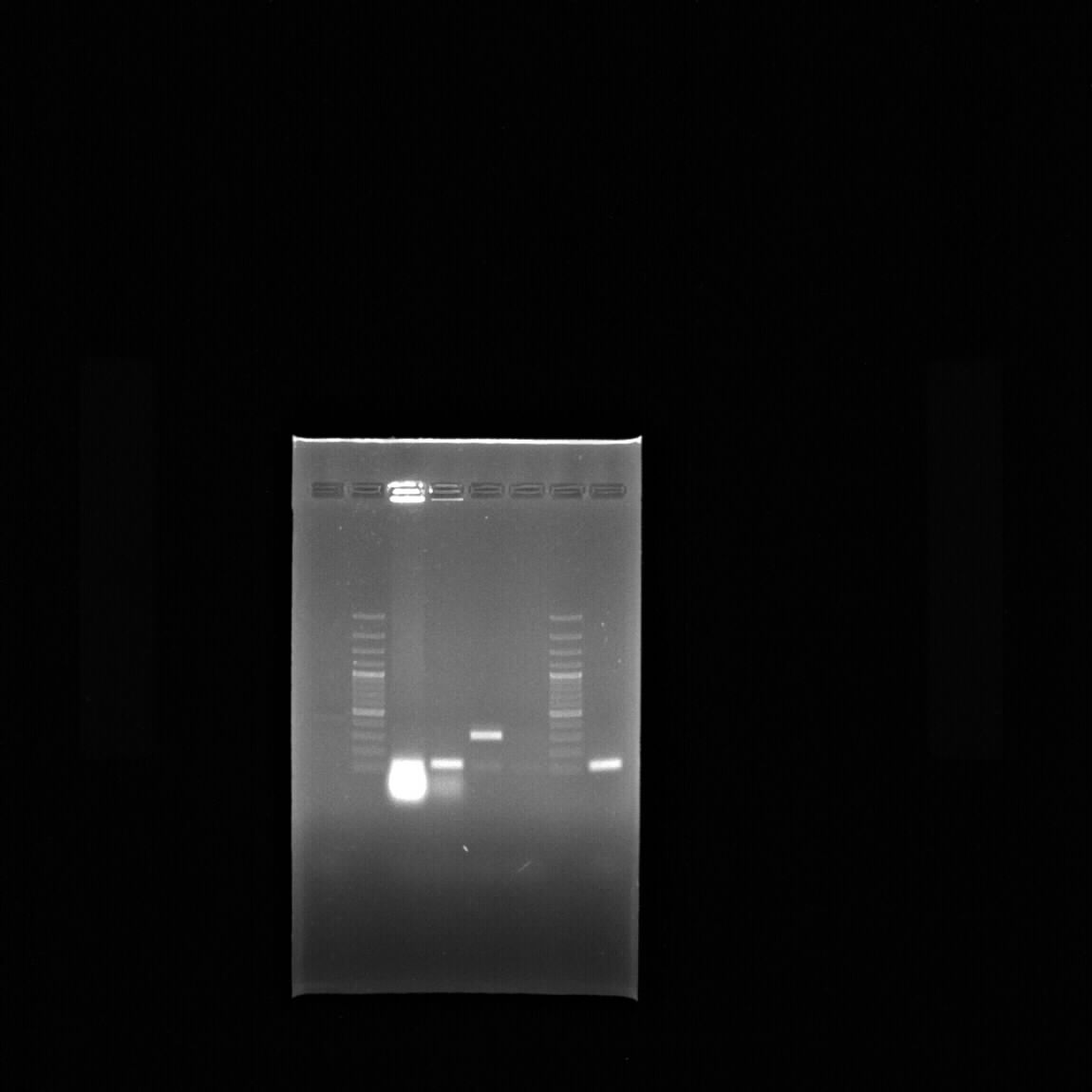

Supplement: Supplementary file 7 — Data S7. Supporting Information. [file PLD3-9-e70034-s007.jpg]
