# Supplementary material for: Modification of Gene Expression Involved in Alkaloid Production in Opium Poppy by VIGS Combined With Pretreatment of Macerozyme Enzyme
Source: Plant Direct. 2025 Jan 7;9(1):e70034. doi: 10.1002/pld3.70034 (PMC11706800; doi:10.1002/pld3.70034)
Supplement: Supplementary file 8 — Table S1. Supporting Information. Table S2. Primers used to quantify gene expression levels. [file PLD3-9-e70034-s008.docx]

**Supplementals:**

**Table 1.**

**a)** Primers used for sequence amplification and ligation into pTRV2

| **Name of primer** | **Target gene** | **Sequences** |
| --- | --- | --- |
| PS_V2_F | *CODM* | GCGCGGATCCGCTCTCAATCGCGACAT |
| PS_V2_R |  | GCGCCTCGAGCAACCGTGATTACATCAC |
| PS_V3_F | *CODM* | GCGCGGATCCTGTGCTTAAATTTCGTGGATGAC |
| PS_V3_R |  | GCGCCTCGAGTCACTTGACCCAAACAGAGTC |
| PS_T6ODM_1F | *T6ODM* | GCGCGGATCCACCTCTACCTAATGCGTTCG |
| PS_T6ODM_1R |  | GCGCCTCGAGTTCCTCCACAAGATCCCCAT |
| PS_T6ODM_2F * | *T6ODM* | GCGCGGATCCCCTTGTCCTCAACCAAAT |
| PS_T6ODM_2R * |  | GCGCCTCGAGTCCACTTTTAAACAAAGC |
| PS_DIOX2_F | *DIOX2* | GCGCGGATCCAGAGTCGGAAATAGGCCCAA |
| PS_DIOX2_R |  | GCGCCTCGAGAAGACGGGGAAAGGACAGTT |

(*Ref from Hagel, J., Facchini, P. Dioxygenases catalyze the O-demethylation steps of morphine biosynthesis in opium poppy. Nat Chem Biol 6, 273–275 (2010). <https://doi.org/10.1038/nchembio.317>)

**b)** Primers used for the detection of positive colonies in pTRV2 and pTRV1

| **Name of primer** | **Target** | **Sequences** |
| --- | --- | --- |
| TRV2_CONT_F: | pTRV2 | GTAAGGTTACCGAATTCTCTAG |
| TRV2_CONT_R: |  | GAACCGTAGTTTAATGTCTTCG |
| TRV1_CONT_F: | pTRV1 | CTTGAAGAAGAAGACTTTCGAAGTCTC |
| TRV1_CONT_R: |  | GTAAAATCATTGATAACAACACAGACAAAC |

**Table 2:** Primers used to quantify gene expression levels**.**

| **Name of primer** | **Target gene** | **Sequences** |
| --- | --- | --- |
| RT_CorF:* | *COR*  (XM_026542858.1) | GCGCGGATCCCCTTGTCCTCAACCAAAT |
| RT_CorR:* |  | GCGCCTCGAGTCCACTTTTAAACAAAGC |
| RT_T6odmF: | *T6ODM*  (GQ500139.1) | TGGAAGGATTTGGACAAGGCT |
| RT_T6odmR: |  | TCTCGGCTGCTTGTACTTGT |
| RT_DioxF: | *DIOX2*  (GQ500140.1) | ACGAACGCATTAGGTAGAGGT |
| RT_DioxR: |  | ACGAACGCATTAGGTAGAGGT |
| RT_CODMF: | *CODM*  (GQ500141.1) | ACGAACGCATTAGGTAGAGGT |
| RT_CODMR: |  | CGACGCTACGGTAAATCCCA |
| PS_Actin_F:* | *PsACT1*  (AB574417) | CACACTGTGCCAATCTATGAG |
| Ps_Actin_R:* |  | TGGATACCAGCAGCTTCCATC |

**(*** Ref from Kawano, N., Kiuchi, F., Kawahara, N., & Yoshimatsu, K. (2012). Genetic and Phenotypic Analyses of a Papaver somniferum T-DNA Insertional Mutant with Altered Alkaloid Composition. Pharmaceuticals, 5(2), 133. <https://doi.org/10.3390/PH5020133>)
