# Supplementary material for: Modification of Gene Expression Involved in Alkaloid Production in Opium Poppy by VIGS Combined With Pretreatment of Macerozyme Enzyme
Source: Plant Direct. 2025 Jan 7;9(1):e70034. doi: 10.1002/pld3.70034 (PMC11706800; doi:10.1002/pld3.70034)
Supplement: Supplementary file 1 — Data S1. Peer Review. [file PLD3-9-e70034-s002.pdf]

Plant Direct
Open Access

MODIFICATION OF GENE EXPRESSION INVOLVED IN ALKALOID PRODUCTION IN OPIUM POPPY BY VIGS COMBINED WITH PRE-TREATMENT OF MACEROZYME ENZYME

Deniz Ece ÖZCAN[1,2], Deniz KÖM[1], Ozan DOĞAN[3], Semiha ERİŞEN[2], Selma ONARICI[1] *

Decision Letter Round 1:

November 11, 2024

Dr. SELMA ONARICI
Tubitak Marmara Arastirma Merkezi
TUBİTAK Marmara Research Center
BKTM Building
Gebze
Kocaeli, N/A
Turkey

RE: MODIFICATION OF GENE EXPRESSION INVOLVED IN ALKALOID PRODUCTION IN OPIUM POPPY BY VIGS COMBINED WITH PRE-TREATMENT OF MACEROZYME ENZYME

Dear Dr. ONARICI:

Thank you for submitting to Plant Direct. All required reviews have been returned and we have now finished our evaluation of your manuscript. In light of the reviewers' and editor's comments, further revisions are needed before the paper can be accepted for publication in Plant Direct.

Please view the editors' and reviewers' comments below and use their suggestions as a guide while you work on your revision.

When uploading the revised version of this article, please be sure to include the following:

-A word document that contains your response to the reviewers. You should respond to each reviewer comment and note the changes made to the manuscript. If you do not agree with a reviewer's comment and choose not to make a suggested revision, please explain why. Please try to provide as complete an answer as possible to each reviewer's criticisms.
-A tracked changes document with each change highlighted
- A clean version of the latest version of the manuscript

To upload your revision, please click the link below.
https://plantdirect.msubmit.net/cgi-bin/main.plex?el=A1Lr4kk4A2oyk1I7A9ftdlZ8RWhbhT7qUsbZC9GXA4gZ

Plant Direct
Open Access

In order to provide as timely a service as possible, we ask that your revision is resubmitted within three months after receipt of this request. If an extension is needed, please send a request, along with a brief explanation, to the editorial office at plantdirect@wiley.com .

Please note that, in addition to publishing reviewer comments, the author's responses to review comments will also be published alongside the final version of the paper. If you would not like the author's responses to be published, please contact the editorial office at plantdirect@wiley.com .

Thank you very much for giving us an opportunity to review your work. I look forward to receiving the next version.

Sincerely,

Yan Lu

Esther van der Knaap

Editor, Plant Direct

--- Editor comments:

--- Reviewer comments:
Reviewer #1:

The manuscript titled "Modification of Gene Expression Involved in Alkaloid Production in Opium Poppy by VIGS Combined with Pre-Treatment of Macerozyme Enzyme" explores the potential of altering secondary metabolite production in opium poppy through the Virus-Induced Gene Silencing (VIGS) technique. This study specifically targets the silencing of genes associated with morphine biosynthesis. Expression levels of four key genes-CODM, T6ODM, COR, and DIOX2-involved in morphine biosynthesis confirm the effectiveness of gene silencing via the VIGS vectors designed for this purpose, along with the application of the macerozyme enzyme.
The study's objectives are novel and clearly outlined, with appropriate methods and methodology. The results are well-presented, and the findings are clearly discussed, highlighting their relevance. This research holds significant promise for future studies focused on gene expression regulation using the VIGS method.

Comments and Suggestions:
1. Abstract Section: The abstract lacks specific findings. The authors should consider including more detailed results to give a clearer overview of the study's outcomes.
2. Macerozyme Introduction: One of the novel aspects of this research is the use of macerozyme during transformation. However, the authors did not provide background information on macerozyme in the introduction section. Details about what macerozyme is and its mechanism of action in transformation should be included.

3. Scientific Name Formatting: In the term "Papaver somniferum L.," the "L." should not be italicized.
4. Abbreviations for Treatments: The abbreviations for the treatments shown in Figure 4 should also appear in Table 2 to improve clarity.
5. Line 261: Please review and correct "2ΔΔCt" with reference to the method by Livak & Schmittgen (2001).
6. Line 339: Verify and correct the result reported as "...causing an 82% decrease," as Figure 4 indicates this should be "72%."
7. Line 346: Confirm and amend "However, V2, V3, D, and V2 applications..." to "However, V2, V3, D, and V2D applications..." to ensure consistency.

Once these issues and comments are addressed, this manuscript will be suitable for publication in Plant Direct.

Reviewer #2:

Ozcan et al. demonstrated simultaneous silencing of benzylisoquinoline alkaloids (BIAs) biosynthesis genes in somatic embryo suspension cultures of opium poppy by Agrobacterium-mediated transformation. To transform the cells with enhanced efficiency, they pre-treated the explants with macerozyme, an enzyme with a known function that has been used for protoplast transformation (https://pmc.ncbi.nlm.nih.gov/articles/PMC7952925/).

The story is incomplete and immature to be published with the data provided in this manuscript. Almost all data provided as main figures and tables can only be considered supplementary data, except the gene expression heatmap (Figure 4). And even on the heatmap, no statistical significance was demonstrated...The datasets provided in Tables 1 and 2, as well as the data shown in Figures 1, 2, and 3, do not show any findings or contain any novel information. Figures 1 and 3 are supplementary, and data shown in Figure 2 is not even required for this study...A basic alignment was sufficient to meet their requirements. Quite a few phylogenetic trees showing the distribution of the same genes are already available in the literature (check Farrow et al., 2013, https://www.sciencedirect.com/science/article/pii/S0021925820488738?via%3Dihub).

The most critical point missing in this work is the explanation of what is the purpose of using the cell suspension culture of opium poppy. Opium poppy cell cultures do not accumulate alkaloids constitutively...If any of the VIGS treatments cause enhancement of any BIAs in cell culture, the value of this research can increase...but no LC-MS data showing the level of BIAs is provided....The differential expression of genes after VIGS treatment can not be attributed to a change in metabolic level adequately without solidly demonstrating the metabolic analysis results...

What was the aim of silencing those genes? It was not well addressed at the beginning of the manuscript...This should be clearly expressed in the abstract as well as in the introduction sections...One would demonstrate the function of the macerozyme in transformation by transiently expressing a marker gene with a visual phenotype (GUS, FiLUC, GFP, etc.) as well...instead, authors choose to perform VIGS hoping to alter the expression of genes on morphine biosynthesis, but no results were provided on if the VIGS treatment caused accumulation of some important BIAs. Overall, the manuscript does not have a single acceptable Figure, and it doesn't meet the standards of publication at this current stage. I advise rejecting this manuscript and suggest authors submitting their work to another journal that is more fitting only after completing their story.

Plant Direct
Open Access

Reviewer #3:

good work

Decision Letter Round 2:

December 2, 2024

Dr. SELMA ONARICI
Tubitak Marmara Arastirma Merkezi
TUBİTAK Marmara Research Center
BKTM Building
Gebze
Kocaeli, N/A
Turkey

RE: MODIFICATION OF GENE EXPRESSION INVOLVED IN ALKALOID PRODUCTION IN OPIUM POPPY BY VIGS COMBINED WITH PRE-TREATMENT OF MACEROZYME ENZYME

Dear Dr. ONARICI:

Thank you for submitting to Plant Direct. All required reviews have been returned and we have now finished our evaluation of your manuscript. In light of the reviewers' and editor's comments, further revisions are needed before the paper can be accepted for publication in Plant Direct.

Please view the editors' and reviewers' comments below and use their suggestions as a guide while you work on your revision.

When uploading the revised version of this article, please be sure to include the following:

-A word document that contains your response to the reviewers. You should respond to each reviewer comment and note the changes made to the manuscript. If you do not agree with a reviewer's comment and choose not to make a suggested revision, please explain why. Please try to provide as complete an answer as possible to each reviewer's criticisms.
-A tracked changes document with each change highlighted
- A clean version of the latest version of the manuscript

Major comments:
Page 6, Line 252-259, Table 2. Table 2 is still confusing. Consider adding "CODM" in the first empty cell in the first column, adding "T6ODM" in the second empty cell in the first column, if they are correct target genes. Considering adding" "CODM+DIOX2" in the first empty cell in the fourth column.

Page 19, Table 1a. Should "PS_DIOX2_F" in the last cell of the first column be "PS_DIOX2_R" instead?

Please also double check the sequences of the forward and reverse primers for DIOX2.

Minor comments:
Page 1, Line 21-23. Grammar problems in this sentence. Consider changing it to:
"To address the cell wall barrier, a known limitation in genetic manipulation, macerozyme pretreatment was employed, significantly enhancing gene silencing efficacy.

Page 1, Line 25 and other places. Consider changing "quantitative real-time PCR" to "quantitative reverse transcription PCR" here and in other places. This proposed change will indicate that the qPCR analysis was performed on cDNA, not DNA. BTW, "real-time" is somewhat redundant to "quantitative".
Page 1, Line 27. Please explain (or spell out) the T1D combination here in the Abstract.

Page 1, Line 29. Please explain (or spell out) the V2 treatment here in the Abstract.

Page 1, Line 31. Please explain (or spell out) the V3D treatment here in the Abstract.

Page 1, Line 42. Consider changing "real-time PCR" to "reverse transcription PCR".

Page 3, Line 125. Consider changing "One of the news in the research" to "One example".

Page 3, Line 133. Consider deleting "structure" after "cell wall permeability".

Page 3, Line 134-136. Please cite some relevant references for this statement.

Page 3, Line 134. Consider changing "grow" to "increase".

Page 5, Line 212. Consider inserting "shown in" before "Table 1".

Page 5, Line 236-237. Consider changing "20g/L", "0.25M", and "10mM" to "20 g/L", "0.25 M", and "10 mM", respectively.

Page 5, Line 236. Please explain what "MS-E" means (E = enzyme?) here.

Page 5, Line 237. "2" in "CaCl2" should be a subscript.

Page 6, Line 261. Consider changing "Real-time quantitative PCR" to "Quantitative reverse transcription PCR".

Page 6, Line 270-271. Consider changing "RT-PCR" to "quantitative PCR" here.

Page 7, Line 297. Change "were performed" to "was performed".

Page 20, Table 2. Consider replacing the two asterisks in the last two cells of the first column with one asterisk, as these two primers were designed by the same authors/reference as "RT_CorF" and "RT_CorR". Please also update the footnote accordingly.

Plant Direct
Open Access

To upload your revision, please click the link below.
https://plantdirect.msubmit.net/cgi-bin/main.plex?el=A7Lr7kk7B1oyk5I4A9ftdlZ8RWhbhT7qUsbZC9GXA4gZ

In order to provide as timely a service as possible, we ask that your revision is resubmitted within three months after receipt of this request. If an extension is needed, please send a request, along with a brief explanation, to the editorial office at plantdirect@wiley.com .

Please note that, in addition to publishing reviewer comments, the author's responses to review comments will also be published alongside the final version of the paper. If you would not like the author's responses to be published, please contact the editorial office at plantdirect@wiley.com .

Thank you very much for giving us an opportunity to review your work. I look forward to receiving the next version.

Sincerely,

Yan Lu

Esther van der Knaap

Editor, Plant Direct

-- Editor comments:
Major comments:
Page 6, Line 252-259, Table 2. Table 2 is still confusing. Consider adding "CODM" in the first empty cell in the first column, adding "T6ODM" in the second empty cell in the first column, if they are correct target genes. Considering adding" "CODM+DIOX2" in the first empty cell in the fourth column.

Page 19, Table 1a. Should "PS_DIOX2_F" in the last cell of the first column be "PS_DIOX2_R" instead? Please also double check the sequences of the forward and reverse primers for DIOX2.

Minor comments:
Page 1, Line 21-23. Grammar problems in this sentence. Consider changing it to:
"To address the cell wall barrier, a known limitation in genetic manipulation, macerozyme pretreatment was employed, significantly enhancing gene silencing efficacy.

Page 1, Line 25 and other places. Consider changing "quantitative real-time PCR" to "quantitative reverse transcription PCR" here and in other places. This proposed change will indicate that the qPCR analysis was performed on cDNA, not DNA. BTW, "real-time" is somewhat redundant to "quantitative".
Page 1, Line 27. Please explain (or spell out) the T1D combination here in the Abstract.

Page 1, Line 29. Please explain (or spell out) the V2 treatment here in the Abstract.

Page 1, Line 31. Please explain (or spell out) the V3D treatment here in the Abstract.

Page 1, Line 42. Consider changing "real-time PCR" to "reverse transcription PCR".

Page 3, Line 125. Consider changing "One of the news in the research" to "One example".

Page 3, Line 133. Consider deleting "structure" after "cell wall permeability".

Page 3, Line 134-136. Please cite some relevant references for this statement.

Page 3, Line 134. Consider changing "grow" to "increase".

Page 5, Line 212. Consider inserting "shown in" before "Table 1".

Page 5, Line 236-237. Consider changing "20g/L", "0.25M", and "10mM" to "20 g/L", "0.25 M", and "10 mM", respectively.

Page 5, Line 236. Please explain what "MS-E" means (E = enzyme?) here.

Page 5, Line 237. "2" in "CaCl2" should be a subscript.

Page 6, Line 261. Consider changing "Real-time quantitative PCR" to "Quantitative reverse transcription PCR".

Page 6, Line 270-271. Consider changing "RT-PCR" to "quantitative PCR" here.

Page 7, Line 297. Change "were performed" to "was performed".

Page 20, Table 2. Consider replacing the two asterisks in the last two cells of the first column with one asterisk, as these two primers were designed by the same authors/reference as "RT_CorF" and "RT_CorR". Please also update the footnote accordingly.

Decision Letter Round 3:

December 6, 2024

Dr. SELMA ONARICI
Tubitak Marmara Arastirma Merkezi
TUBİTAK Marmara Research Center
BKTM Building
Gebze
Kocaeli, N/A
Turkey

MSID: 2024-01620R2
MS TITLE: MODIFICATION OF GENE EXPRESSION INVOLVED IN ALKALOID PRODUCTION IN OPIUM POPPY

Plant Direct
Open Access

BY VIGS COMBINED WITH PRE-TREATMENT OF MACEROZYME ENZYME

Dear Dr. SELMA ONARICI:

I am pleased to inform you that your manuscript "MODIFICATION OF GENE EXPRESSION INVOLVED IN ALKALOID PRODUCTION IN OPIUM POPPY BY VIGS COMBINED WITH PRE-TREATMENT OF MACEROZYME ENZYME" has been accepted for publication in Plant Direct.

Your article will appear online in the next available issue of Plant Direct. To ensure your article gets published as quickly as possible, please pay attention to the steps detailed below. We have found that most of the delays happen at this stage, especially at the payment stage, so please respond as quickly as possible when prompted.

License Agreement: Once your article has been accepted it will move to Production and undergo admin and file checking - you may receive an email with any queries we have at this stage. When all required items are received by the publisher and queries resolved, the corresponding author will receive an email from Wiley's Author Services system which will ask them to log on at https://authorservices.wiley.com/bauthor and will present them with the appropriate license for completion. Your article cannot be published until both the signed license agreement and payment of the article fee have been received.

Payment of the Open Access Article Publication Fee: All articles published in Plant Direct are fully open access: immediately and freely available to read, download and share. Plant Direct charges a publication fee to cover the publication costs. The corresponding author for this manuscript should have already received a quote from the payments team (cs-openaccess@wiley.com) with the estimated article publication fee; please email cs-openaccess@wiley.com if this has not been received. The corresponding author should log on to the Wiley Author Services site, where the publication fee can be paid by credit card or an invoice. Pro Forma can also be requested. Payment of the publication charge must be received before the article will be published online.

Proofs: You will have the opportunity to look over your paper once more when you receive the author proofs for your article. The proofs will be with you in approximately two weeks. Please note that, in addition to publishing reviewer comments, the author's responses to review comments will also be published alongside the final version of the paper. If you would not like the author's responses to be published, please contact the editorial office at plantdirect@wiley.com .

Promotion of your article: You can help your research get the attention it deserves! Check out Wiley's free Promotion Guide for best-practice recommendations for promoting your work at www.wileyauthors.com/eeo/guide. And learn more about Wiley Editing Services which offers professional video, design, and writing services to create shareable video abstracts, infographics, conference posters, lay summaries, and research news stories for your research at www.wileyauthors.com/eeo/promotion.

Thank you again for your contribution to Plant Direct. If you have any questions, feel free to contact the editorial office at plantdirect@wiley.com .

Sincerely,

Yan Lu

Esther van der Knaap

Editor, Plant Direct

Response to Reviewer Round 1:

Response to Reviewer #1 Comments:
Thank you for your valuable comment. We have carefully considered your suggestion and have revised the manuscript accordingly.

Comment 1: **Abstract Section: The abstract lacks specific findings. The authors should consider including more detailed results to give a clearer overview of the study's outcomes.**

Response 1: In line with your suggestions, the abstract section has been rewritten and arranged to reflect the study and its results more clearly and effectively.

Comment 2: **Macerozyme Introduction: One of the novel aspects of this research is the use of macerozyme during transformation. However, the authors did not provide background information on macerozyme in the introduction section. Details about what macerozyme is and its mechanism of action in transformation should be included.**

Response 2: An explanatory text about the Macerozyme enzyme, provided below, has been added between lines 131 and 143 in tracked copy.

Besides these, various methodologies have been developed, among which enzymatic aid for genetic transformation is considered a breakthrough. One of the news in the research is the utilization of maceration enzymes during the transformation. Macerozyme, a cell wall-degrading enzyme of fungal origin, is produced mainly for protoplast isolation in plant tissue cultures. This enzyme breaks down cell wall components, including cellulose and pectin, allowing protoplasts to be released (Ramesh et al., 2023). Similarly, some other studies have determined that the treatment with macerating enzymes improved Agrobacterium-mediated transformation efficiency in plant cultures (Du et al., 2019; Kumar et al., 2006; Weber et al., 2003). These studies represent the capability of the enzyme to allow the entry of foreign DNA into plant cells by increasing the cell wall permeability structure, and after digestion, the area where Agrobacterium can bind to plant cells may grow. In addition, breakdown or weakening of the cell wall may result in the release of chemicals capable of activating bacterial viral genes, hence increasing the efficiency of genetic transformation.

Comment 3: **Scientific Name Formatting: In the term "Papaver somniferum L.," the "L." should not be italicized.**

Response 3: The entire manuscript has been reviewed, and the italic formatting of "L" in "Papaver somniferum L." has been corrected.

Comment 4. **Abbreviations for Treatments: The abbreviations for the treatments shown in Figure 4 should also appear in Table 2 to improve clarity.**

Response 4. To improve the correlation between Figure 4 and Table 2, and to enhance the comprehensibility of the applications within the manuscript, two additional columns have been added to Table 2, incorporating the abbreviation tags used in the Results section. The description of Table 2 has also been revised accordingly following this addition.

Comment 5. **Line 261: Please review and correct "2ΔΔCt" with reference to the method by Livak & Schmittgen (2001).**

Response 5: The formatting error in "2ΔΔCt" has been corrected, line 281 in tracked copy.

<u>Comment 6.</u> **Line 339: Verify and correct the result reported as "...causing an 82% decrease," as Figure 4 indicates this should be "72%."**
Response 6: Line 337 in tracked copy: Thank you for your attention. The typo has been corrected.
<u>Comment 7</u>**. Line 344: Confirm and amend "However, V2, V3, D, and V2 applications..." to "However, V2, V3, D, and V2D applications..." to ensure consistency.**
Response 7: Line 338 in draft copy: The phrase "However, V2, V3, D, and V2 applications..." has been revised to "However, V2, V3, D, and V2D applications..."

We appreciate your insightful suggestion, which has helped improve the clarity of our manuscript

Response to Reviewer #2 Comments:
We have carefully examined Reviewer 2's comments and deemed it appropriate to provide the following responses to the suggestions.
<u>Comment 1:</u> ***To transform the cells with enhanced efficiency, they pre-treated the explants with macerozyme, an enzyme with a known function that has been used for protoplast transformation.***
Response 1: As you pointed out, the removal of cell walls is a prerequisite for generating and stably transforming protoplast cultures, and this is not a novel concept. However, the aim of this study was not to achieve genetic transformation stably but to establish a methodology for applying Virus-Induced Gene Silencing (VIGS), a widely used tool in plant functional genetics. This work aims to pave the way for successful implementation of VIGS to somatic cell cultures of opium poppy.
<u>Comment 2:</u> ***The datasets provided in Tables 1 and 2, as well as the data shown in Figures 1, 2, and 3, do not show any findings or contain any novel information.***
Response 2:

- Table 1 provides the names of the genes and vector pairings used in the study. In studies where vectors targeting specific genes are created, presenting this information is critical for ensuring proper analysis of target-interaction-outcome relationships for readers.
- Table 2 contains a dataset outlining the transformation combinations applied in the study. Without this dataset, it would be impossible to understand to readers which applications targeted which gene(s), leading to incomplete information in the text.
- Figure 1 offers a brief overview of how somatic embryo cultures were generated from the target organism, the opium poppy. Nowhere in the manuscript do we claim that generating these cultures is a novel achievement.
- Figure 2 summarizes bioinformatic analysis data obtained during the design process for the primary modification tool, the vectors. We believe that understanding the similarities among target genes is vital for designing effective constructs.
- Figure 3 presents the inserts which cloned into vectors specifically designed for this study, <u>contributing to the uniqueness of the research</u>.

<u>Comment 3:</u> ***The most critical point missing in this work is the explanation of the purpose of using the cell suspension culture of opium poppy. Opium poppy cell cultures do not accumulate alkaloids constitutively... If any of the VIGS treatments cause enhancement of any BIAs in cell culture, the value of this research can increase...***
Response 3: In the Discussion section of our manuscript, we cited Tam et al. (1980), who demonstrated the active involvement of the BIA pathway in opium poppy somatic cell cultures. We have also validated

this in a separate preliminary study, wherein the Ofis1 genotype used as the genetic source produced BIA levels comparable to those observed under field conditions (approximately 1% of fresh weight). However, due to confidentiality agreements with the commissioning organization, we could not share the complete dataset from our preliminary analyses.

Comment 4: ***But no LC-MS data showing the level of BIAs is provided... The differential expression of genes after VIGS treatment cannot be attributed to a change in metabolic level adequately without solidly demonstrating the metabolic analysis results...***

Response 4: Indeed, we did not present LC-MS or HPLC data in this study. The primary objective of this research was to investigate the genetic pathway rather than optimize large-scale production systems. VIGS, as a transient transformation method, does not provide statistically robust results within a few hours. Since this study focuses on functional genetic analysis rather than altering alkaloid production, we deemed LC-MS or HPLC analysis unnecessary. Moreover, we do not claim in the manuscript that we altered alkaloid production. However, we cited Alagoz et al. (2016), who reported similar manipulations using stable transformations and presented chemical analysis results in their study.

Comment 5: ***What was the aim of silencing those genes? It was not well addressed at the beginning of the manuscript... This should be clearly expressed in the abstract as well as in the introduction sections... One would demonstrate the function of the macerozyme in transformation by transiently expressing a marker gene with a visual phenotype (GUS, FiLUC, GFP, etc.) as well... Instead, the authors choose to perform VIGS hoping to alter the expression of genes on morphine biosynthesis, but no results were provided on if the VIGS treatment caused accumulation of some important BIAs.***

Response 5: We acknowledge that there were shortcomings in the initial version of the abstract, which we have revised following recommendations from reviewers. It is not an arbitrary attempt but a systematically designed effort to establish the foundation for functional genetic studies. As noted earlier, our aim was not to develop stable transformation methods but to establish a functional framework for further investigations.

We sincerely thank you for your valuable insights.

Response to Reviewer #3 Comments:

We greatly appreciate your valuable support.

Response to Reviewer Round 2:

We sincerely thank you for all the suggestions provided to enhance the quality and clarity of the manuscript. The following list includes all the revisions you requested. We have thoroughly reviewed them and implemented the desired corrections.

While incorporating the three references you requested into the tracked document, a systematic issue arose with the Mendeley Reference Manager software. As a result, we were unable to use the track changes function for these specific additions. However, the corresponding sections where these references were included have been highlighted in yellow, as we did with all other corrections.

Funding information was added to the main document.

Below, you will find a detailed list of our responses to each of your suggestions and the actions taken to address them.

Kind regards,

Selma ONARICI

Added references:

Pitzschke, A. (2013). Agrobacterium infection and plant defense-transformation success hangs by a thread. Frontiers in Plant Science, 4(DEC), 70185. https://doi.org/10.3389/FPLS.2013.00519/BIBTEX
Gelvin, S. B. (2014). Traversing the cell: AgrobacteriumT-DNA's journey to the host genome. Frontiers in Plant Science, 3(MAR), 21986. https://doi.org/10.3389/FPLS.2012.00052/BIBTEX
Zaltsman, A., Krichevsky, A., Kozlovsky, S. V., Yasmin, F., & Citovsky, V. (2010). Plant defense pathways subverted by Agrobacterium for genetic transformation. Plant Signaling & Behavior, 5(10), 1245–1248. https://doi.org/10.4161/PSB.5.10.12947

Major comments:

- ***Page 6, Line 252-259, Table 2. Table 2 is still confusing. Consider adding "CODM" in the first empty cell in the first column, adding "T6ODM" in the second empty cell in the first column, if they are correct target genes. Considering adding" "CODM+DIOX2" in the first empty cell in the fourth column.***

Response: The table has been revised as per your request. The expressions CODM, T6ODM, and CODM+DIOX have been added to the relevant cells.

- ***Page 19, Table 1a. Should "PS_DIOX2_F" in the last cell of the first column be "PS_DIOX2_R" instead? Please also double check the sequences of the forward and reverse primers for DIOX2.***

Response: The typographical error you mentioned has been corrected. The sequences have been verified against databases and the datasets we utilized, ensuring their accuracy.

Minor comments:

- ***Page 1, Line 21-23. Grammar problems in this sentence. Consider changing it to: "To address the cell wall barrier, a known limitation in genetic manipulation, macerozyme pretreatment was employed, significantly enhancing gene silencing efficacy.***

Response: The sentence in text has been revised according to your recommendation.

- ***Page 1, Line 25 and other places. Consider changing "quantitative real-time PCR" to "quantitative reverse transcription PCR" here and in other places. This proposed change will indicate that the qPCR analysis was performed on cDNA, not DNA. BTW, "real-time" is somewhat redundant to "quantitative".***
- ***Page 1, Line 42. Consider changing "real-time PCR" to "reverse transcription PCR".***
- ***Page 6, Line 270-271. Consider changing "RT-PCR" to "quantitative PCR" here.***
- ***Page 6, Line 261. Consider changing "Real-time quantitative PCR" to "Quantitative reverse transcription PCR".***

Response: The revisions you suggested have been implemented throughout the entire manuscript.

- ***Page 1, Line 27. Please explain (or spell out) the T1D combination here in the Abstract.***
- ***Page 1, Line 29. Please explain (or spell out) the V2 treatment here in the Abstract.***

- ***Page 1, Line 31. Please explain (or spell out) the V3D treatment here in the Abstract.***

Response: The abbreviations T1D, V2, and V3D have been updated to T6ODM+DIOX, CODM, and CODM+DIOX, respectively.

- ***Page 3, Line 125. Consider changing "One of the news in the research" to "One example".***
- ***Page 3, Line 133. Consider deleting "structure" after "cell wall permeability***".

Response: The sentences in text have been revised according to your recommendation.

- ***Page 3, Line 134-136. Please cite some relevant references for this statement.***

Response: Following relevant references were added fort he statement.

- Pitzschke, A. (2013). Agrobacterium infection and plant defense-transformation success hangs by a thread. Frontiers in Plant Science, 4(DEC), 70185. https://doi.org/10.3389/FPLS.2013.00519/BIBTEX
- Gelvin, S. B. (2014). Traversing the cell: AgrobacteriumT-DNA's journey to the host genome. Frontiers in Plant Science, 3(MAR), 21986. https://doi.org/10.3389/FPLS.2012.00052/BIBTEX
- Zaltsman, A., Krichevsky, A., Kozlovsky, S. V., Yasmin, F., & Citovsky, V. (2010). Plant defense pathways subverted by Agrobacterium for genetic transformation. Plant Signaling & Behavior, 5(10), 1245–1248. https://doi.org/10.4161/PSB.5.10.12947

- ***Page 3, Line 134. Consider changing "grow" to "increase".***
- ***Page 5, Line 212. Consider inserting "shown in" before "Table 1".***
- ***Page 5, Line 236-237. Consider changing "20g/L", "0.25M", and "10mM" to "20 g/L", "0.25 M", and "10 mM", respectively.***

Response: All of the corrections you suggested have been applied.

- ***Page 5, Line 236. Please explain what "MS-E" means (E = enzyme?) here.***

Response: Yes, that abbreviation is used in reference to the enzyme.

- ***Page 5, Line 237. "2" in "CaCl2" should be a subscript.***
- ***Page 7, Line 297. Change "were performed" to "was performed".***

Response: All of the corrections you suggested have been applied.

- ***Page 20, Table 2. Consider replacing the two asterisks in the last two cells of the first column with one asterisk, as these two primers were designed by the same authors/reference as "RT_CorF" and "RT_CorR". Please also update the footnote accordingly.***

Response: The asterisk errors you mentioned have been corrected.
